# Use of Cardiopulmonary Exercise Testing to Evaluate Long COVID-19 Symptoms in Adults: A Systematic Review and Meta-analysis

**DOI:** 10.1001/jamanetworkopen.2022.36057

**Published:** 2022-10-12

**Authors:** Matthew S. Durstenfeld, Kaiwen Sun, Peggy Tahir, Michael J. Peluso, Steven G. Deeks, Mandar A. Aras, Donald J. Grandis, Carlin S. Long, Alexis Beatty, Priscilla Y. Hsue

**Affiliations:** 1 Department of Medicine, University of California, San Francisco; 2 Division of Cardiology, Zuckerberg San Francisco General Hospital, San Francisco, California; 3 UCSF Library, University of California, San Francisco; 4 Division of HIV, Infectious Diseases, and Global Medicine, Zuckerberg San Francisco General Hospital, University of California, San Francisco; 5 Division of Cardiology, UCSF Health, University of California, San Francisco; 6 Department of Epidemiology and Biostatistics, University of California, San Francisco

## Abstract

**Importance:**

Reduced exercise capacity is commonly reported among individuals with COVID-19 symptoms more than 3 months after SARS-CoV-2 infection (long COVID-19 [LC]). Cardiopulmonary exercise testing (CPET) is the criterion standard to measure exercise capacity and identify patterns of exertional intolerance.

**Objectives:**

To estimate the difference in exercise capacity among individuals with and without LC symptoms and characterize physiological patterns of limitations to elucidate possible mechanisms of LC.

**Data Sources:**

A search of PubMed, EMBASE, Web of Science, preprint servers, conference abstracts, and cited references was performed on December 20, 2021, and again on May 24, 2022. A preprint search of medrxiv.org, biorxiv.org, and researchsquare.com was performed on June 9, 2022.

**Study Selection:**

Studies of adults with SARS-CoV-2 infection more than 3 months earlier that included CPET-measured peak oxygen consumption (V̇o_2_) were screened independently by 2 blinded reviewers; 72 (2%) were selected for full-text review, and 35 (1%) met the inclusion criteria. An additional 3 studies were identified from preprint servers.

**Data Extraction and Synthesis:**

Data extraction was performed by 2 independent reviewers according to the PRISMA reporting guideline. Data were pooled using random-effects models.

**Main Outcomes and Measures:**

Difference in peak V̇o_2_ (in mL/kg/min) among individuals with and without persistent COVID-19 symptoms more than 3 months after SARS-CoV-2 infection.

**Results:**

A total of 38 studies were identified that performed CPET on 2160 individuals 3 to 18 months after SARS-CoV-2 infection, including 1228 with symptoms consistent with LC. Most studies were case series of individuals with LC or cross-sectional assessments within posthospitalization cohorts. Based on a meta-analysis of 9 studies including 464 individuals with LC symptoms and 359 without symptoms, the mean peak V̇o_2_ was −4.9 (95% CI, −6.4 to −3.4) mL/kg/min among those with symptoms with a low degree of certainty. Deconditioning and peripheral limitations (abnormal oxygen extraction) were common, but dysfunctional breathing and chronotropic incompetence were also described. The existing literature was limited by small sample sizes, selection bias, confounding, and varying symptom definitions and CPET interpretations, resulting in high risk of bias and heterogeneity.

**Conclusions and Relevance:**

The findings of this systematic review and meta-analysis study suggest that exercise capacity was reduced more than 3 months after SARS-CoV-2 infection among individuals with symptoms consistent with LC compared with individuals without LC symptoms, with low confidence. Potential mechanisms for exertional intolerance other than deconditioning include altered autonomic function (eg, chronotropic incompetence, dysfunctional breathing), endothelial dysfunction, and muscular or mitochondrial pathology.

## Key Points

**Question:**

Is exercise capacity reduced more than 3 months after SARS-CoV-2 infection among those with long COVID-19 (LC) symptoms compared with recovered individuals without symptoms, and what patterns of limitations on cardiopulmonary exercise testing (CPET) are common?

**Findings:**

In this systematic review and meta-analysis of 38 studies comprising 2160 participants, exercise capacity was reduced by 4.9 mL/kg/min among individuals with symptoms consistent with LC compared with individuals without symptoms more than 3 months after SARS-CoV-2 infection. Findings among individuals with exertional intolerance suggest that deconditioning, dysfunctional breathing, chronotropic incompetence, and abnormal peripheral oxygen extraction and/or use may contribute to reduced exercise capacity.

**Meaning:**

These findings suggest that CPET may provide insight into the mechanisms for reduced exercise capacity among individuals with LC.

## Teaser

This systematic review and meta-analysis addresses whether adults with persistent COVID-19 symptoms more than 3 months after SARS-CoV-2 infection (long COVID-19 [LC]) have reduced exercise capacity compared with recovered individuals without symptoms and identifies potential mechanisms of LC.

## Introduction

After SARS-CoV-2 infection, a substantial proportion of survivors with long COVID-19 (LC) experience persistent cardiopulmonary symptoms and exercise intolerance. Long COVID-19 may occur in 3% to 30% of individuals after SARS-CoV-2 infection,^1,2,3,4,5^ including nonhospitalized and vaccinated individuals,^6,7^ and can persist for at least 12 months.^8^

Cardiopulmonary exercise testing (CPET) is the criterion standard for measuring exercise capacity and aiding in the differential diagnosis of exercise limitations.^9,10,11^ After measuring resting cardiopulmonary parameters, participants exercise on a cycle ergometer or a treadmill with measurement of gas exchange and cardiopulmonary monitoring. Measuring oxygen consumption (V̇o_2_) allows for objective and reproducible determination of exercise capacity, determination of anaerobic threshold, and classification of limitations. Research CPET has provided insight into persistent symptoms after SARS,^12^ dyspnea in people living with HIV,^13^ and exercise intolerance in myalgic encephalitis and/or chronic fatigue syndrome (ME/CFS).^14,15,16^ Clinically, CPET is useful diagnostically for unexplained dyspnea^9^ and prognostically in heart failure,^17^ lung disease,^9^ and preoperative evaluations.^18^

Case series suggest that SARS-CoV-2 infection is associated with reduced exercise capacity.^19,20^ A prior narrative review of 11 studies including 581 patients^21^ suggested that deconditioning was a major cause of reduced exercise capacity after COVID-19 hospitalization, with literature limited by confounding and lack of controls; however, to our knowledge, no systematic review on the role of CPET in LC has been published. Whether exercise intolerance persists and is associated with LC and the pathophysiology of exertional intolerance in LC, especially among individuals who are not hospitalized, is uncertain. Therefore, the objectives of this systematic review and meta-analysis were to address whether adults with persistent COVID-19 symptoms more than 3 months after SARS-CoV-2 infection^22^ have reduced exercise capacity on results of CPET compared with recovered individuals without symptoms and to identify potential causal pathways for the reduced exercise capacity after SARS-CoV-2 infection.

## Methods

This systematic review and meta-analysis followed the Preferred Reporting Items for Systematic Reviews and Meta-analyses (PRISMA) reporting guideline and was registered prospectively on PROSPERO (CRD42021299842) before beginning the literature search. Because the study did not constitute human participants research, the University of California, San Francisco Institutional Review Board deemed it exempt from approval and waived informed consent.

We included studies that performed CPET measurement of peak V̇o_2_ among adults at least 3 months after SARS-CoV-2 infection, including case series (only symptomatic individuals), cohort studies (both symptomatic and recovered individuals), and baseline data from interventional studies. Of 3256 studies that were screened by title and abstract, we selected 72 (2%) for full-text review, (1%) of which met the inclusion criteria. Studies were excluded if participants were studied less than 3 months after infection or if they did not measure V̇o_2_. For the first objective, we only included studies that compared individuals with and without prevalent symptoms consistent with LC at the time of CPET; for the second objective, we included studies that classified participants with exercise limitations or explored specific mechanisms of limitations.

A comprehensive search was planned with a research librarian (P.T.) to identify all studies that used CPET to evaluate exercise capacity among adults more than 3 months after SARS-CoV-2 infection, including studies published since 2020, abstracts from conference proceedings, and indexed preprints without language restrictions. We searched PubMed, EMBASE, Web of Science, and references of included studies. We additionally searched medrxiv.org, biorxiv.org, and researchsquare.com for nonindexed preprints. The search strategy included terms and synonyms for the following: *COVID* or *SARS-CoV-2* along with *cardiopulmonary exercise test*, *CPET* or *CPX* or *CPEX*, *exercise capacity*, *V̇o_2_*, and *anaerobic threshold* tailored to each search engine (eTable 1 in the Supplement). Searches were conducted on December 20, 2021, and rerun on May 24, 2022; the preprint search was performed on June 9, 2022.

The searches were conducted by the research librarian (P.T.), with results downloaded and imported into a commercially available systemic reviews tool. After duplicates were automatically removed, 2 independent reviewers (M.S.D. and K.S.) screened each title and abstract using the systemic reviews tool and were blinded to each other’s decision regarding full-text review. Studies for which both reviewers agreed to full-text review or disagreed after reconciliation discussion underwent full-text review. After full-text review and consensus discussion, there were no disagreements regarding study inclusion. Data extraction was performed independently, in duplicate, using REDCap (eMethods and eAppendix in the Supplement). Discrepancies were corrected by the first 2 authors (M.S.D. and K.S.) reviewing the full text together.

Quality was assessed independently by 2 reviewers (M.S.D. and K.S.) using Cochrane’s Quality in Prognostic Studies tool^23^ to assess study populations (inclusion criteria and control group), measurement quality (CPET exercise protocols, peak V̇o_2_ assessment, submaximal test results, and interpretation of CPET), outcome (symptom assessment), confounding, and statistical analysis and reporting, followed by discussion and tabulation of consensus results. The GRADE (Grading of Recommendations, Assessment, Development, and Evaluations) framework was used to guide a consensus discussion for overall outcome assessment.^24^

Owing to expected differences in study inclusion criteria, we used random-effects meta-analysis to estimate the mean difference in peak V̇o_2_ (in mL/kg/min) between those with and without prevalent LC symptoms as defined by each study using a restricted maximum likelihood variance estimator and Wald-type confidence intervals. For 2 studies that only reported median and IQR, the distance between the median and IQR upper and lower bounds were similar, so medians were taken as the mean and SD was estimated as the IQR divided by 1.35^25^; subgroups were combined among studies only reporting results by groups.^25^ Heterogeneity was assessed by examining forest plots, funnel plots, heterogeneity variance (τ^2^ statistic), and inconsistency (*I*^2^ statistic). Prespecified subgroup analyses by proportion hospitalized and time since infection were performed. Because of the small number of studies, tests for publication bias were not performed.

To synthesize findings for our second objective, we recorded the predominant explanatory finding for reduced exercise capacity, including deconditioning, ventilatory limitation, cardiac limitations, chronotropic incompetence, dysfunctional breathing/ventilatory inefficiency, or other limitations, and the number and proportion with each if reported. Meta-analysis was performed using Stata, version 17.1 (StataCorp LLC); 2-sided *P* < .05 indicated statistical significance.

## Results

**Figure 1.**
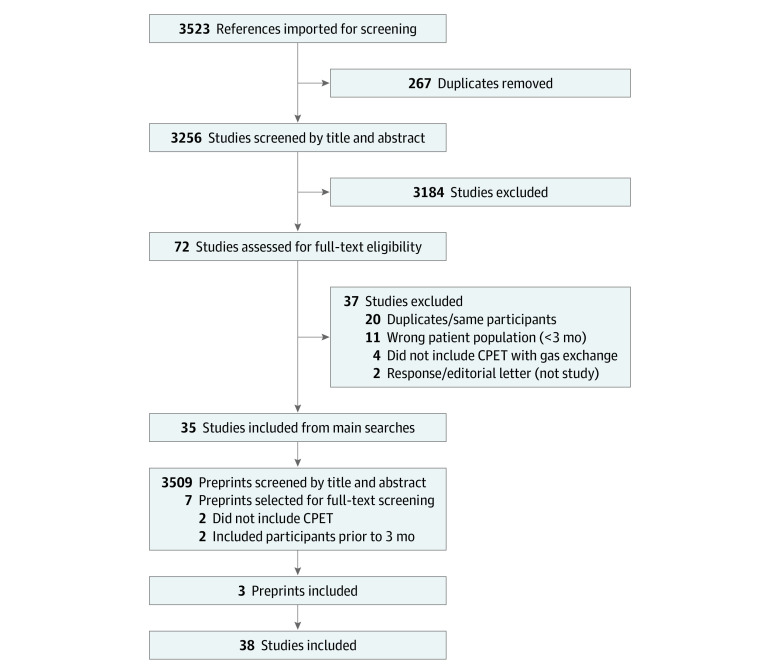
Study Screening Diagram Among 3523 references identified through our primary searches, we identified 72 studies for full-text review, of which 35 met the inclusion criteria. We additionally screened 3509 preprints and identified 7 for full-text review, resulting in a total of 38 studies included. CPET indicates cardiopulmonary exercise testing.

We identified 41 reports of 38 observational studies in which CPET was performed among 2160 individuals 3 to 18 months after SARS-CoV-2 infection,^26,27,28,29,30,31,32,33,34,35,36,37,38,39,40,41,42,43,44,45,46,47,48,49,50,51,52,53,54,55,56,57,58,59,60,61,62,63,64,65,66^ including 1228 individuals with symptoms consistent with LC. The studies included 33 published reports, 2 conference abstracts, and 3 preprints (Figure 1). We identified 1 interventional study of cardiac rehabilitation^67^ with baseline CPET reported in an included study.^68^

### Description of Included Studies

Table 1 lists study-level characteristics; eTable 2 and eTable 3 in the Supplement include the risk of bias assessments. Most studies (32 [84.2%]) were single-center case series of patients attending LC clinics or referred for clinical CPET (only symptomatic individuals) or cross-sectional assessments within COVID-19 recovery cohorts (with and without current symptoms). Most studies performed CPET 3 to 6 months after infection; only 1 study^26^ investigated individuals more than 1 year after infection. Fourteen studies (36.8%)^27,30,33,35,36,44,47,49,52,55,56,62,66,68^ only included individuals who were hospitalized for acute infection (median, 70% [range, 0-100%] hospitalized), and 15 studies (39.5%)^30,32,39,40,41,42,43,50,51,54,55,57,59,61,64^ included individuals with prevalent symptoms at CPET (median, 95% [range, 38%-100%] symptomatic).

**Table 1.** Summary of Studies That Included Cardiopulmonary Exercise Testing (CPET) More Than 3 Months After SARS-CoV-2 Infection

Source	Report type	Study design, sampling, and/or recruitment	No. with SARS-CoV-2 infection	Hospitalized with infection, No./total No. (%)	Prevalent symptoms consistent with LC, No./total No. (%)	Time since infection, d^a^	Primary analytic comparisons
Abdallah et al,^45^ 2021	Research letter	Prospective cohort	63	25/63 (40)	34/49 (69)	125 (16)	Hospitalized vs nonhospitalized^45^ and fatigue vs no fatigue^58^^b^
Alba et al,^42^ 2021	Peer-reviewed published report	Retrospective cohort referred for CPET from LC clinic	18	3/18 (17)	18/18 (100)	257.5 (149-322)	PASC vs controls
Ambrosino et al,^55^ 2022	Peer-reviewed published report	Pulmonary rehabilitation after severe COVID-19	36	36/36 (100)	36/36 (100)	NR	Normal vs reduced exercise capacity
Aparisi et al,^47^ 2021	Peer-reviewed published report	Prospective cohort post hospitalization	70	70/70 (100)	41/70 (59)	181 (42)	Persistent dyspnea vs no residual dyspnea
Barbagelata et al,^38^ 2022	Peer-reviewed published report	Retrospective EHR review of individuals referred for clinical CPET	200	39/200 (20)	112/200 (56)	80 (21)	LC vs no LC
Blumberg et al,^28^ 2022	Preprint	Cross-sectional study	43	NR	NR	119 (24)	Vaccinated vs unvaccinated
Borrego Rodriguez et al,^59^ 2021	Conference abstract	Nonhospitalized health care workers	57	0	57/57 (100)	>90	Peak V̇o_2_ >100% vs <100% of predicted levels
Brown et al,^44^ 2022	Peer-reviewed published report	Prospective hospitalized cohort without ICU stay, myocardial injury, or comorbidities	40	40/40 (100)	20/40 (50)	Median, 106	Self-reported normal exercise capacity vs reduced exercise capacity vs controls
Cassar et al,^27^ 2021	Peer-reviewed published report	Prospective cohort after COVID hospitalization	42	42/42 (100)	NR (89% overall)	180 (180-204)	Change in CPET from 2-3 mo to 6 mo and vs controls
Clavario et al,^68^ 2021	Peer-reviewed published report	Prospective cohort after COVID hospitalization	200	200/200 (100)	160/200 (80)	107 (83-189)	Normal vs reduced exercise capacity
de Boer et al,^40^ 2022	Research letter	Retrospective case series of clinically referred for CPET	50	5/50 (10)	50/50 (100)	180 (120)	Fatty acid and lactate production in PASC vs published cohorts
Debeaumont et al,^30^ 2021	Peer-reviewed published report	Retrospective case series of hospitalized patients with COVID-19 referred for CPET	23	23/23 (100)	23/23 (100)	180	Ward vs ICU
Dorelli et al,^49^ 2021	Research letter	Prospective cohort post hospitalization without comorbidities	28	28/28 (100)	NR	169 (28)	Exercise ventilatory inefficiency vs efficiency
Durstenfeld et al,^26^ 2022	Preprint	Prospective cohort without cardiovascular disease	39	7/39 (18)	23/39 (59)	525 (465-552)	Cardiopulmonary symptoms vs no symptoms
Evers et al,^54^ 2022	Peer-reviewed published report	Retrospective case series of patients referred for post–COVID-19 exercise limitation or dyspnea	30	21/30 (70)	30/30 (100)	Mean, 129	Change from CPET assessment 1 to 2
Frésard et al,^50^ 2022	Peer-reviewed published report	Retrospective cohort of clinical CPET among patients referred for LC and persistent dyspnea	51	36/51 (71)	51/51 (100)	119 (89)	Dysfunctional breathing vs normal breathing
Godinho and Freeman,^61^ 2021	Conference abstract	Case series of nonhospitalized patients with persistent exercise limitations	9	0	9/9 (100)	Range, 180-360	Descriptive
Jahn et al,^62^ 2021	Research letter	Case series of patients with severe COVID-19 pneumonitis attending posthospitalization pulmonary rehabilitation	35	35/35 (100)	NR	90	Impaired vs normal peak V̇o_2_
Johnsen et al,^63^ 2021	Peer-reviewed published report	Case series of post–COVID-19 clinic referrals for CPET for symptoms	31	NR (60% overall)	NR (67% overall)	90	Nonhospitalized vs hospitalized
Kersten et al,^53^ 2021	Peer-reviewed published report	Case series of post–COVID-19 clinic referrals for CPET if initial test results not revealing	36	NR (8% overall)	NR	121 (77)	Descriptive
Ladlow et al,^31^ 2022	Peer-reviewed published report	Prospective cohort of active military personnel	113	35/87 (31)	61/87 (70)	159 (7)	Comparisons by hospitalization and persistent symptoms compared with controls
Liu et al,^52^ 2021	Peer-reviewed published report	Prospective posthospitalization cohort	41	41/41 (100)	NR	219 (11)	Pulmonary fibrosis vs no fibrosis
Mancini et al,^43^ 2021	Peer-reviewed published report	Case series of LC clinic referrals for CPET for symptoms	41	9/41 (22)	41/41 (100)	267 (99)	Descriptive
Margalit et al,^37^ 2022	Peer-reviewed published report	Nested case-control study within COVID recovery cohort	141	14/141 (10)	66/141 (47)	240 (75)	Fatigue vs no significant fatigue
Mohr et al,^41^ 2021	Research letter	Case series of post–COVID-19 clinic referrals for CPET for dyspnea	10	6/10 (60)	10/10 (100)	Mean, 115	Descriptive
Motiejunaite et al,^46^ 2021	Research letter	Prospective cohort	114	104/114 (91)	58/114 (51)	90 (71-106)	DLCO >75 vs ≤75
Moulson et al,^57^ 2022	Peer-reviewed published report	Case series of young athletes referred for symptoms	21	NR	21/21 (100)	90 (63)	Young symptomatic athletes vs historical controls
Parkes et al,^64^ 2021	Preprint	Retrospective cohort of patients undergoing clinical CPET	12	9/12 (75)	12/12 (100)	182 (111)	Descriptive
Pleguezuelos et al,^56^ 2021	Peer-reviewed published report	Survivors of ARDS due to bilateral COVID-19 pneumonia requiring mechanical ventilation and tracheostomy	15	15/15 (100)	NR	NR	Mechanical efficiency, peak V̇o_2_, and power output in patients with COVID-19 vs 3 control groups
Ribeiro Baptista et al,^36^ 2022	Peer-reviewed published report	Prospective cohort with severe COVID-19 requiring hospitalization >7 d and oxygen	105	105/105 (100)	NR	90 d after discharge	Normal vs reduced exercise capacity
Rinaldo et al,^35,65^ 2021	Research letter	Prospective cohort post hospitalization	75	75/75 (100)	39/75 (52)	97 (26)	Normal vs reduced exercise capacity
Romero-Ortuno et al,^32^ 2022	Peer-reviewed published report	Cross-sectional study of symptomatic individuals within a prospective cohort	80	14/80 (17)	80/80 (100)	Median, 320 (range, 39-655)	Attaining >85% of predicted maximum heart rate
Singh et al,^39^ 2022	Peer-reviewed published report	Prospective cohort referred for CPET from LC clinic for unexplained exercise intolerance with negative initial findings of workup	10	1/10 (10)	10/10 (100)	330 (30)	LC vs controls
Skjørten et al,^33^ 2021	Peer-reviewed published report	Multicenter prospective cohort post hospitalization	156	156/156 (100)	59/156 (38)	104 (90-139)	COVID-19 vs reference population norms and no dyspnea (mMRC, 0) vs dyspnea (mMRC, 1-4)
Szekely et al,^29^ 2021	Peer-reviewed published report	Prospective cohort of individuals evaluated at the emergency department for acute COVID-19	71	NR	48/71 (68)	91 (26)	COVID-19 vs control; asymptomatic vs symptomatic; severity of acute illness
Vannini et al,^66^ 2021	Research letter	Prospective cohort post hospitalization	41	41/41 (100)	29/41 (71)	180	Severity of acute illness and peak V̇o_2_ <80% vs ≥80%
von Gruenewaldt et al,^51^ 2022	Peer-reviewed published report	Retrospective cohort of clinical CPET	20	8/20 (40)	20/20 (100)	217 (133-329)	Normal vs abnormal breathing pattern
Vonbank et al,^34^ 2021	Peer-reviewed published report	Prospective cohort	100	18/100 (18)	NR	Median, 112	Severity of acute infection

Abbreviations: ARDS, adult respiratory distress syndrome; DLCO, diffusion capacity of carbon monoxide; EHR, electronic health record; ICU, intensive care unit; LC, long COVID-19; mMRC, modified Medical Research Council Dyspnea Scale; NR, not reported; PASC, postacute sequelae of COVID-19; V̇o_2_, oxygen consumption.

^a^ Typically defined as the time from symptom onset or positive results of polymerase chain reaction testing; some studies used the date of admission or date of hospital discharge. Unless indicated otherwise, data are presented as mean (SD) or median (IQR). Pleguezuelos et al^56^ reported time since hospital discharge and mean hospitalization of 23 days but not time from infection to hospitalization; therefore, it was unclear whether infection occurred more than 3 months previously. We identified an additional study by Ladlow et al^69^; however, because it seemed likely that there were overlapping participants, we only included the main study.

^b^ Report on same prospective cohort study.

### Exercise Capacity in Symptomatic Compared With Recovered Individuals

**Figure 2.**
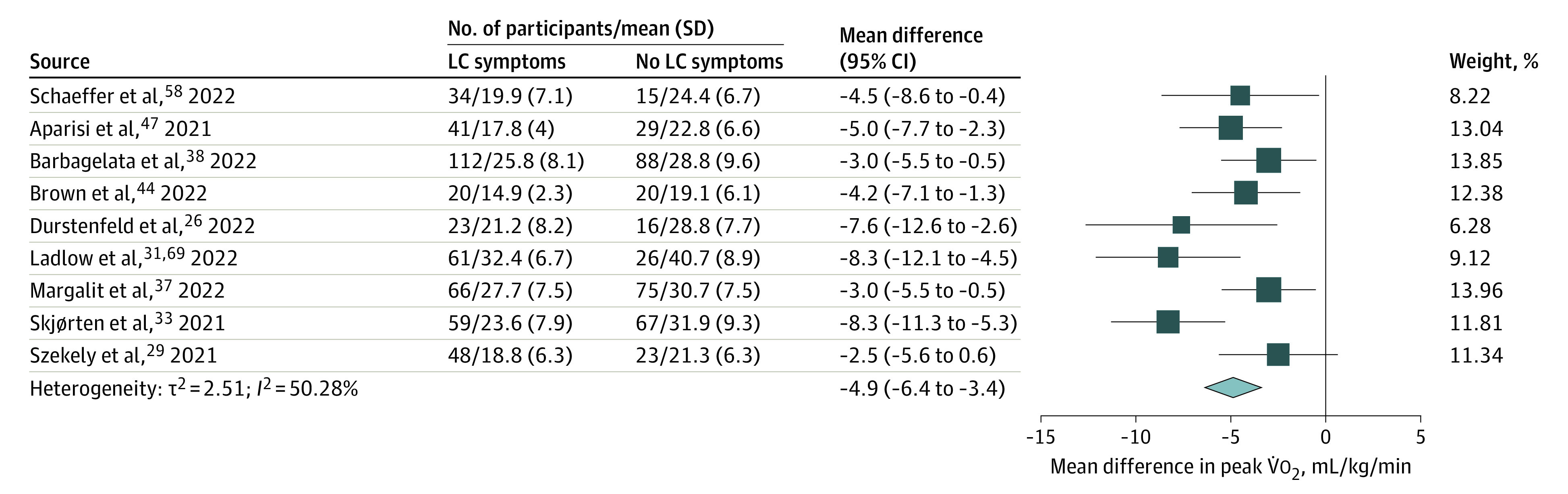
Meta-analysis of Peak Oxygen Consumption (V̇o_2_) Among Studies Comparing Patients With and Without Long COVID-19 (LC) Symptoms By random-effects meta-analysis of 9 studies that included 464 individuals with LC symptoms and 359 individuals without LC symptoms (as defined by each study), the mean difference in peak V̇o_2_ was −4.9 (95% CI, −6.4 to −3.4) mL/kg/min.

Nine studies^26,29,31,33,37,38,44,45,47,58^ included both individuals with prevalent symptoms (n = 464) and recovered individuals without prevalent symptoms (n = 359) (Table 2), with the risk of bias rated for each study in eTable 2 in the Supplement and overall quality in the eResults in the Supplement. Because definitions of LC and postacute sequelae of COVID-19 have evolved, studies used different symptom definitions, mostly based on prevalent symptoms at CPET (dyspnea, fatigue, or exertional intolerance). From meta-analyses of these 9 studies, mean peak V̇o_2_ is estimated to be –4.9 (95% CI, –6.4 to –3.4) mL/kg/min among individuals with symptoms (*P* < .001) (Figure 2). The I^2^ statistic and funnel plot (eFigure 1 in the Supplement) suggest moderate heterogeneity. Two studies that did not find a statistically significant difference in symptom prevalence by reduced or preserved peak V̇o_2_^68^ or by association between improvement in peak V̇o_2_ and symptoms^27^ were excluded for not reporting peak V̇o_2_ by symptoms.

**Table 2.** Studies Reporting Peak Oxygen Consumption (V̇o_2_) Among Individuals With and Without Prevalent Symptoms Consistent With Long COVID-19 (LC) After SARS-CoV-2 Infection^a^

Source	Definition of LC symptoms	Age, y	Sex, No. (%)		BMI	Hospitalized, No. (%)	Time after infection, d	Participants with LC symptoms			Participants with no LC symptoms		
			Female	Male				No.	Peak V̇o_2_, mL/kg/min	Peak V̇o_2_, % predicted	No.	Peak V̇o_2_, mL/kg/min	Peak V̇o_2_, % predicted
Aparisi et al,^47^ 2021	Persistent dyspnea vs no dyspnea	55 (12)	45 (64)	25 (36)	27 (5)	70 (100)	181 (42)	41	17.8 (15.8-21.2)	77.8 (64.0-92.5)	29	22.8 (18.8-27.7)	99 (88-105)
Barbagelata et al,^38^ 2022	Dyspnea or fatigue persisting >45 d after onset	49 (14)	98 (49)	102 (51)	26 (6)	39 (20)	80 (21)	112	25.8 (8.1)	89.7 (19.9)	88	28.8 (9.6)	92.9 (18.7)
Brown et al,^44^ 2022	Self-reported reduced exercise capacity	52	22 (55)	18 (45)	28	40 (100)	106	20	14.9 (13.1-16.2)	NR	20	19.1 (15.4-23.7)	NR
Durstenfeld et al,^26^ 2022	Chest pain, dyspnea, palpitations, or fatigue	52 (42-61)	18 (39)	28 (61)	30	7 (18)	526 (464-553)	23	21.2 (8.2)	89 (23)	16	28.8 (7.7)	111 (20)
Ladlow et al,^31^ 2022	≥1 Symptom	39	13 (15)	74 (85)	29	35 (31)	159 (7)	61	32.4 (6.7)	NR	26	40.7 (8.9)	NR
Margalit et al,^37^ 2022	Fatigue	47 (13)	83 (59)	58 (41)	28 (5)	14 (14)	240 (75)	66	27.7 (7.5)	96.1 (18.3)	75	30.7 (7.5)	99.6 (17.4)
Schaeffer et al,^58^ 2022	Fatigue vs no fatigue	48	23 (47)	26 (53)	29	25 (40)	125 (16)	34	19.9 (7.1)	74 (20)	15	24.4 (6.7)	81 (17)
Skjørten et al,^33^ 2021	Dyspnea, mMRC, 1-4 vs 0	56	60 (38)	96 (62)	28 (5)	156 (100)	104 (90-139)	59	23.6 (7.9)	NR	67	31.9 (9.3)	NR
Szekely et al,^29^ 2021	Persistent fatigue, dyspnea, muscle weakness, or pain	53 (16)	24 (34)	47 (66)	28 (6)	NR^b^	91 (26)	48	18.8 (6.3) and 1.5 (0.5) L/min	NR	23	21.3 (6.3) and 1.7 (0.5) L/min	NR

Abbreviations: BMI, body mass index (calculated as weight in kilograms divided by height in meters squared); mMRC, modified Medical Research Council Dyspnea Scale; NR, not reported.

^a^ Unless indicated otherwise, data are presented as mean (SD) or median (IQR). Cells with single values report the mean; SDs were only reported by subgroup.

^b^ All patients were evaluated in the emergency department, but the number or percentage admitted was not reported.

Clinical and methodologic variability likely contribute to heterogeneity. Clinical variability may result from the spectrum of LC severity and symptoms.^70^ Most studies recruited before widespread vaccination, but 1 study^28^ reported lower peak V̇o_2_ among unvaccinated compared with vaccinated individuals. In addition, methodologic variability in the definition of LC^26,29^ and CPET exercise modality (treadmill, upright cycle ergometer, or supine cycle ergometer) may also contribute. Most studies suggest higher acuity during acute infection (intensive care unit–treated vs hospitalized vs nonhospitalized patients) is associated with worse exercise capacity,^30,31,32,33,34^ although this is not a universal finding.^35,36^ Subgroup analyses by proportion hospitalized or time after SARS-CoV-2 infection were not significantly different compared with the overall result (eResults in the Supplement).

Residual confounding may also contribute to heterogeneity. Age, sex, body mass index, and prior fitness are highly associated with peak V̇o_2_ and likely associated with LC, but few studies addressed confounding. Margalit et al^37^ reported that age, sex, pre–COVID-19 fitness, body mass index, and reduction in exercise time per week were similar among those with and without LC. Barbagaleta et al^38^ adjusted for sex, cardiovascular history, use of β-blockers, and use of aspirin but not body mass index or age, and mean estimated peak V̇o_2_ was 3.2 (95% CI, 0.9-5.5) mL/min/kg lower in LC. Durstenfeld et al^26^ estimated that mean peak V̇o_2_ was 5.9 (95% CI, 2.3-9.6) mL/kg/min lower in LC adjusted for age, sex, body mass index, time since infection, and hospitalization.

### Patterns of Reduced Exercise Capacity

We included 37 studies with at least 714 people with reduced exercise capacity that classified patterns or limitations or investigated specific mechanisms (Table 3).^26,27,29,30,31,33,34,35,36,37,38,39,40,41,42,43,44,45,46,47,48,49,50,51,52,53,54,55,56,57,58,59,60,61,62,63,64,65,66,68,69^ Nearly all studies defined reduced exercise capacity as less than 80% or less than 85% of predicted levels. Approaches to CPET interpretation may differ, but few studies reported using specific guidelines or algorithms or their classification approach, so notable differences emerge between studies even with similar objective findings.

**Table 3.** Patterns of Limitations Among Individuals With Reduced Exercise Capacity More Than 3 Months After SARS-CoV-2 Infection

Source	Reduced exercise capacity, No. (%)	Pattern of limitations^a^						Other mechanisms
		Deconditioning	Peripheral	Cardiac	Ventilatory	Chronotropic incompetence	Dysfunctional breathing and/or ventilatory inefficacy	
Abdallah et al,^45^ 2021	41 (65)	Other identified	Other identified	NR	NR	Primary	NR	NR
Alba et al,^42^ 2021	6 (33)	NA	Other identified	Other identified	Not identified	NR	NR	NR
Ambrosino et al,^55^ 2022	28 (78)	Other identified	Other identified	NR	NR	NR	Other identified	Endothelial dysfunction
Aparisi et al,^47^ 2021	NR	NR	NA	NR	NR	NR	Primary	NA
Barbagelata et al,^38^ 2022	39 (35)	NR	Other identified	Primary	Other identified	NR	NR	NR
Borrego Rodriguez et al,^59^ 2021	32 (56)	Primary	Other identified	NA	Not identified	NR	NR	NR
Brown et al,^44^ 2022	20 (50)	NR	Not identified	Primary	Not identified	NR	NR	Preload failure
Cassar et al,^27^ 2021	6 (19)	Primary	Other identified	Not identified	Not identified	NR	NR	Symptom limitation (submaximal tests)
Clavario et al,^68^ 2021	99 (50)	Other identified	Other identified	Other identified	Other identified	NR	NR	NR
de Boer et al,^40^ 2022	16 (32)	NR	Primary	Other identified	Not identified	NR	NR	Altered metabolism
Debeaumont et al,^30^ 2021	12 (52)	Other identified	NR	NR	Not identified	NR	Primary	NR
Dorelli et al,^49^ 2021	NR	NR	NR	NR	NR	NR	Primary	NR
Durstenfeld et al,^26^ 2022	15 (38)	Other identified	NA	Other identified	Not identified	Primary	NR	NR
Evers et al,^54^ 2022	11 (37)	NR	Primary	NR	NR	NR	NR	NR
Frésard et al,^50^ 2022	NR	NR	Other identified	NR	Primary (s)	NR	Primary (m)	NR
Godinho and Freeman,^61^ 2021	5 (50)	Primary	Other identified	NR	Not identified	NR	NR	NR
Jahn et al,^62^ 2021	19 (54)	Primary	NR	Other identified	Other identified	NR	NR	NR
Johnsen et al,^63^ 2021	16 (52)	Primary	NR	NR	Other identified	NR	NR	NR
Kersten et al,^53^ 2021	17 (55)	Other identified	NR	Other identified	Other identified	NR	NR	Pulmonary vascular
Ladlow et al,^31^ 2022	4 (7)	Other identified (s)	Other identified (m)	NR	NR	NR	Other identified (m)	NR
Liu et al,^52^ 2021	NR	NR	NR	NR	NR	NR	Other identified	Pulmonary fibrosis
Mancini et al,^43^ 2021	24 (59)	NR	Other identified	Other identified	Not identified	NR	Primary	Preload failure, pulmonary hypertension
Margalit et al,^37^ 2022	NR	NR	NR	NR	NR	Primary	NR	NR
Mohr et al,^41^ 2021	8 (80)	NR	Primary	Other identified	Other identified	NR	NR	Critical illness polyneuropathy
Motiejunaite et al,^46^ 2021	86 (75)	Primary	NR	Not identified	Other identified	NR	Primary	“Lack of motivation” (submaximal tests)
Moulson et al,^57^ 2022	3 (14)	NR	NR	Not identified	Primary	Other identified	NR	Exertional hypotension
Parkes et al,^64^ 2021	10 (83)	Primary	NR	NR	Other identified	NR	Primary	Pulmonary vascular
Pleguezuelos et al,^56^ 2021	NR	NR	Primary	NR	NA	NR	NR	Mechanical inefficiency
Ribeiro Baptista et al,^36^ 2022	37 (35)	Primary	NR	Not identified	Other identified	NR	NR	NR
Rinaldo et al,^35,65^ 2021	41 (55)	Primary	Other identified	NR	Not identified	NR	NR	NR
Singh et al,^39^ 2022	NR	NR	Primary	Not identified	NR	NR	Primary	NR
Skjørten et al,^33^ 2021	49 (31)	Primary	NR	Other identified	Other identified	NR	Other identified	NR
Szekely et al,^29^ 2021	49 (69)	NA	Not identified	Other identified	Not identified	Primary	NR	Insufficient stroke volume increase
Vannini et al,^66^ 2021	19 (46)	Not identified	Not identified	Other identified	Other identified	NR	NR	NR
von Gruenewaldt et al,^51^ 2022	2 (20)	NR	NR	NR	NR	NR	Primary	NR
Vonbank et al,^34^ 2021	NR	NR	Other identified	Other identified	Other identified	NR	NR	NR

Abbreviation: NA, not applicable; NR, not reported.

^a^ Designation (s) indicates a pattern noted among patients with severe acute illness; (m), a pattern noted among nonhospitalized patients with acute COVID-19.

Deconditioning was reported as the most prevalent pattern by 10 studies,^27,33,35,36,46,59,61,62,63,64^ with alterations in muscular oxygen utilization acknowledged as an alternative explanation by some. Eight studies^27,33,35,36,46,62,63,64^ reporting deconditioning included mostly individuals hospitalized for severe acute COVID-19. Deconditioning may be more common among those hospitalized with other patterns (peripheral, ventilatory inefficiency) predominant among nonhospitalized patients.^31^ Muscular and/or peripheral oxygen extraction abnormalities were also commonly reported. Distinguishing deconditioning from altered oxygen delivery, mitochondrial dysfunction, muscular pathology, and obesity can be challenging with noninvasive CPET without adjunctive testing or pre–COVID-19 CPET for comparison. Using invasive CPET, Singh et al^39^ found reduced peripheral oxygen extraction, and others^40,41^ reported alterations in metabolism and lactate production. Importantly, none of the studies that included adjunctive cardiac imaging or right heart catheterization during exercise testing attributed their findings to deconditioning.^29,39,42,43,44^

Cardiac limitations were uncommon, but studies with adjunctive cardiac testing identified reduced stroke volume augmentation that was likely attributable to preload failure^29,43,44^; Singh et al^39^ did not find evidence of preload failure. Five studies^26,29,37,45,69^ identified chronotropic incompetence as a contributor.

Although ventilatory limitations were uncommon, dysfunctional breathing, hyperventilation, or ventilatory inefficiency (V/Q mismatch) were commonly noted.^43,46,47,48,49,50,51^ One study each specifically reported dysautonomia,^69^ pulmonary fibrosis,^52^ pulmonary vascular limitation,^53^ impaired microcirculation,^54^ endothelial dysfunction,^55^ and loss of mechanical efficiency^56^ as the primary cause of reduced exercise capacity. Despite concerns about pulmonary thromboembolism during acute infection, pulmonary vascular limitations were uncommon.

### Longitudinal Trends

Four studies^27,54,57,67^ performed longitudinal CPET in a subset, including 1 interventional study. Cassar et al^27^ reported CPET at 2 to 3 and at 6 months; median peak V̇o_2_ improved from 18.0 (IQR, 14.4-21.9) to 20.5 (IQR, 17.5-26.1) mL/kg/min but remained lower than that of controls (28.1 [95% CI, 22.1-34.0] mL/kg/min; *P* ≤ .001 for all). Evers et al^54^ found no change in peak V̇o_2_ during 3 months among 23 individuals with reduced exercise capacity who underwent repeated CPET (mean [SD] CPET 1: 86% [19%] of predicted levels; CPET 2: 85% [21%] of predicted levels; *P* = .55). Moulson et al^57^ found improved peak V̇o_2_ among young symptomatic athletes 5 months after the index study, which correlated with symptom resolution. Barbara et al^67^ found that mean (SD) peak V̇o_2_ improved from 17.8 (4.6) to 20.5 (4.5) mL/kg/min after 8 weeks of cardiac rehabilitation (*P* < .001).

## Discussion

This meta-analysis and systematic review found 38 studies that reported CPET on 2160 individuals after SARS-CoV-2 infection, including 1228 with prevalent symptoms possibly consistent with LC and 714 with reduced exercise capacity. In our meta-analysis of symptomatic vs recovered individuals more than 3 months after SARS-CoV-2 infection, we found a modest but consistent effect suggesting that exercise capacity was reduced among individuals with LC, with very low certainty in the magnitude of the effect size by GRADE (eResults in the Supplement). Given the low certainty by GRADE, we identified classifications of exercise limitations without a single conclusive mechanism. Despite the large number of participants included, the overall quality of the evidence is poor owing to the small sample size of most studies, selection bias, variability in symptom ascertainment and CPET interpretation, inadequate methods to address confounding, and lack of appropriate statistical methods.

### Challenges to Estimating the Association of LC With Exercise Capacity

Selection bias was a major challenge; the included studies oversampled hospitalized individuals with greater acute severity, more comorbidities, and lower baseline fitness. Hospitalization or need for intensive care during acute infection was associated with reduced peak V̇o_2_ and with LC,^30,31,32,33,34^ but most patients with LC were not hospitalized.^71^ Differential selection bias may occur among individuals who are hospitalized, are referred for clinical CPET, or attend CPET after joining a cohort, which may result in overestimation of the proportion of individuals with reduced exercise capacity.

Few studies addressed confounding; the most commonly used strategies included (1) reporting the percentage of predicted peak V̇o_2_ that implicitly adjusts for age, sex, height, and weight; (2) group matching on age, sex, and weight; and (3) excluding individuals with comorbid cardiac, pulmonary, and musculoskeletal conditions. Preinfection fitness was an unmeasured confounder in all but 1 study^37^; no studies had preinfection CPET to compare within-individual change. Two excluded studies among military recruits and professional athletes found reduced peak V̇o_2_ at 45 to 60 days after infection compared with before infection.^72,73^ A few studies used stepwise regression despite small sample sizes and colinear variables, resulting in exclusion of important confounders. Only 2 studies^26,38^ estimated an adjusted difference in peak V̇o_2_ between individuals with and without LC symptoms.

Using the GRADE framework, we have low confidence in our meta-analysis estimate of the difference in exercise capacity among individuals with and without LC symptoms. The included studies provided evidence of a clinically significant, mild to moderate decrease in exercise capacity among individuals with LC compared with infected individuals without LC symptoms despite different definitions of LC.

### Insights Into Mechanisms of Reduced Exercise Capacity in LC

These studies should provide insight into mechanisms of LC, yet no consistent etiology of reduced exercise capacity has emerged, likely because of heterogeneity in inclusion criteria, variability in interpretation (measurement error), and the presence of multiple mechanisms of reduced exercise capacity in LC. Deconditioning, which occurs to some degree after any illness but especially during and after hospitalization, was commonly identified. On results of noninvasive CPET, peripheral mechanisms related to oxygen delivery and/or extraction due to muscular, mitochondrial, or vascular pathology can be misattributed to deconditioning. Use of invasive CPET, stress echocardiography, or stress magnetic resonance imaging allows for measurement or approximation of cardiac output, preload, pulmonary hypertension, and peripheral oxygen extraction and may therefore allow for more accurate classification. Overall, we found consistent evidence that deconditioning is not the only explanation of reduced exercise capacity in LC, especially among individuals who were not hospitalized.

Apart from peripheral mechanisms, other commonly reported patterns include (1) dysfunctional breathing or hyperventilation unexplained by baseline pulmonary function tests or findings on cross-sectional imaging, (2) chronotropic incompetence, and (3) preload failure despite normal resting cardiac function. Ventilatory, pulmonary vascular, and cardiac limitations are uncommon, suggesting that direct heart or lung damage (especially given other negative testing results) are not major drivers of exercise limitations in LC. From the diversity of interpretations, different phenotypes resulting in exertional intolerance seem more likely than a single unifying mechanism.

Autonomic dysfunction and endothelial dysfunction are possible mechanisms for these findings and could be caused by SARS-CoV-2 infection of neurons and endothelial cells, chronic inflammation, or autoimmune mechanisms. One included study found endothelial dysfunction^55^ and 2 suggested dysautonomia^37,69^ to be associated with reduced exercise capacity in LC. Dysfunctional breathing may also be a manifestation of dysautonomia.^69^ Autonomic nervous system and endothelial interaction may regulate peripheral vasomotor tone^16^; together, they may explain differences in peripheral extraction and preload failure. Small-fiber neuropathy among individuals who have LC symptoms with postural orthostatic tachycardia syndrome may be associated with reduced cerebral blood flow and postural symptoms.^74,75^ No published studies included comprehensive autonomic testing, endothelial testing, and CPET.

### Comparison With ME/CFS

Myalgic encephalitis/chronic fatigue syndrome is associated with reduced peak V̇o_2_, lower ventilatory efficiency, higher perceived exertion, and lower peak heart rates,^15^ and chronotropic incompetence may contribute to exercise limitations.^14^ Alternatively, small-fiber neuropathy causing peripheral shunting reduces exercise capacity in ME/CFS.^16^ Postexertional malaise (PEM; recurrence or worsening of symptoms after exercise) has been reported in LC, similar to ME/CFS.^76,77^ The overlap between ME/CFS and LC and whether LC has similar pathophysiology to ME/CFS remain unknown.

### Recommendations for CPET for LC Clinical Care and Research

Given the heterogeneity of phenotypes of LC and lack of a single mechanism, CPET is clinically useful to narrow the differential diagnosis of exertional dyspnea in LC. A CPET result within reference range without cardiopulmonary limitations will reassure some individuals with LC and increase comfort with physical activity. For those with objective limitations, identifying a cardiac or ventilatory limitation could provide clues for further diagnostic testing and treatment. Risk of PEM should be considered in evaluation of the risk-benefit ratio of CPET among individuals reporting PEM.

With regard to research, determining the prevalence of exercise intolerance requires intentional sampling. Selection of control groups requires particular attention tailored to the research question. We recommend that CPET be performed as a maximal test that allows for assessment of chronotropy except for individuals with significant PEM, with adjunctive measures as per local expertise. Careful postexertional symptom assessment, including after CPET and 2-day CPET protocols, may provide insights into PEM in individuals with LC symptoms. Correlative data with autonomical testing may provide mechanistic insights. Given high reproducibility within individuals and reduced exercise capacity among individuals with LC symptoms, CPET may be a useful objective measure to include in interventional trials for potential LC therapeutics.

### Limitations

This study has some limitations. The search plan was not peer reviewed, and the search was not limited to peer-reviewed studies. We may have missed studies that met our inclusion criteria, especially recent preprints. Many included studies were case series, which contributed only to classification of exercise limitations. Because of selection bias, we could not estimate the prevalence of reduced exercise capacity. There was moderate heterogeneity in the included studies. Additionally, we cannot rule out publication bias contributing to exaggeration of effect estimates, especially because 2 excluded studies did not find an association, although we mitigated this by including preprints and conference abstracts.

## Conclusions

In this meta-analysis and systematic review, we found evidence that exercise capacity is reduced after SARS-CoV-2 infection among individuals who have symptoms consistent with LC, with a low confidence in the effect size. Further research should include longitudinal assessments to understand the trajectory of exercise capacity. Interventional trials of potential therapies are urgently needed, including studies of rehabilitation to address deconditioning, as well as further mechanistic investigation into dysfunctional breathing, autonomic dysfunction, chronotropic incompetence, impaired oxygen uptake or utilization, and preload failure to identify treatments for LC.

## Notes

Supplement. **eTable 1.** Search Strategies for PubMed, Web of Science, and EMBASE **eTable 2.** Quality Assessment and Potential Threats to Validity Among Studies Included in Comparison of Peak V̇o_2_ Among Those With and Without Symptoms >3 Months After SARS-CoV-2 Infection **eTable 3.** Quality Assessment and Potential Threats to Validity Among Studies Included in Assessment of Limitations of Exercise Capacity **eMethods.** Study Protocol **eAppendix.** Study Findings and Quality Form **eResults.** Sensitivity Analyses and GRADE Assessment **eFigure.** Funnel Plot of Studies Comparing Peak V̇o_2_ Among People With and Without Symptoms
