# Supplementary material for: Use of Cardiopulmonary Exercise Testing to Evaluate Long COVID-19 Symptoms in Adults: A Systematic Review and Meta-analysis
Source: JAMA Netw Open. 2022 Oct 12;5(10):e2236057. doi: 10.1001/jamanetworkopen.2022.36057 (PMC9557896; doi:10.1001/jamanetworkopen.2022.36057)
Supplement: Supplement. — eTable 1. Search Strategies for PubMed, Web of Science, and EMBASE eTable 2. Quality Assessment and Potential Threats to Validity Among Studies Included in Comparison of Peak V̇o2 Among Those With and Without Symptoms >3 Months After SARS-CoV-2 Infection eTable 3. Quality Assessment and Potential Threats to Validity Among Studies Included in Assessment of Limitations of Exercise Capacity eMethods. Study Protocol eAppendix. Study Findings and Quality Form eResults. Sensitivity Analyses and GRADE Assessment eFigure. Funnel Plot of Studies Comparing Peak V̇o2 Among People With and Without Symptoms [file jamanetwopen-e2236057-s001.pdf]

Supplementary Online Content

Durstenfeld MS, Sun K, Tahir P, et al. Use of cardiopulmonary exercise testing to evaluate long COVID-19 symptoms in adults: a systematic review and meta-analysis. *JAMA Netw Open*. 2022;5(10):e2236057. doi:10.1001/jamanetworkopen.2022.36057

eTable 1. Search Strategies for PubMed, Web of Science, and EMBASE
eTable 2. Quality Assessment and Potential Threats to Validity Among Studies Included in Comparison of Peak $\dot{V}O_2$ Among Those With and Without Symptoms >3 Months After SARS-CoV-2 Infection
eTable 3. Quality Assessment and Potential Threats to Validity Among Studies Included in Assessment of Limitations of Exercise Capacity
eMethods. Study Protocol
eAppendix. Study Findings and Quality Form
eResults. Sensitivity Analyses and GRADE Assessment
eFigure. Funnel Plot of Studies Comparing Peak $\dot{V}O_2$ Among People With and Without Symptoms

This supplementary material has been provided by the authors to give readers additional information about their work.

© 2022 Durstenfeld MS et al. *JAMA Network Open*.

eTable 1. Search Strategies for PubMed, Web of Science, and EMBASE

DATABASE	SEARCH STRATEGY
PubMed	("COVID-19"[Mesh] OR COVID OR "SARS-CoV-2"[Mesh] OR SARS-CoV-2) AND (“cardiopulmonary exercise test*” OR CPET OR CPX OR CPEX OR exercise capacity OR VO2 OR "Anaerobic Threshold"[Mesh] OR anaerobic threshold)
Web of Science	(COVID OR SARS-CoV-2) AND (“cardiopulmonary exercise test*” OR CPET OR CPX OR CPEX OR exercise capacity OR VO2 OR anaerobic threshold)
Embase	('coronavirus disease 2019'/exp OR 'coronavirus disease 2019') AND ('cardiopulmonary exercise test'/exp OR 'cardiopulmonary exercise test' OR 'cardiopulmonary exercise testing'/exp OR 'cardiopulmonary exercise testing' OR cpet OR cpx OR cpex OR 'exercise capacity'/exp OR 'exercise capacity' OR vo2 OR 'anaerobic threshold'/exp OR 'anaerobic threshold')

© 2022 Durstenfeld MS et al. *JAMA Network Open*.

eTable 2. Quality Assessment and Potential Threats to Validity Among Studies Included in Comparison of Peak $\dot{V}O_2$ Among Those With and Without Symptoms >3 Months After SARS-CoV-2 Infection

First Author, Year	Study Participation	Study Attrition	CPET Protocol, Execution & Interpretation	Assessment of LC Symptoms	Confounding	Statistical Analysis & Reporting	Key Threats to Validity
Aparisi, et al,[51] 2021	**Moderate**<br>Mostly hospitalized	**Moderate**<br>53/522 (10%) of hospitalized and few non-hospitalized	**Moderate**<br>Treadmill ramp<br>Low average RER	**Moderate**<br>Used standardized non-COVID tools for dyspnea	**High**<br>Not addressed	**High**<br>No adjusted models	• Selection Bias<br>• Low average RER suggests submaximal CPET<br>• Confounding<br>• Lack of interpretation of individual studies
Barbagelata et al,[41], 2021	**High**<br>Retrospective EHR-based study without explanation for why individuals without LC underwent CPET	**Moderate**<br>No information provided	**High**<br>Treadmill with individualized Bruce/ modified Bruce<br>High proportion low RER studies	**High**<br>Defined as dyspnea or fatigue >45 days after symptom onset but ascertained through chart review	**Moderate**<br>Adjust for gender, cardiovascular history, and use of beta blockers	**Moderate**<br>Data-driven variable selection	• Retrospective EHR-based study without clarity regarding comparison group of people without LC—why CPETS were performed on 88 individuals "without LC" at exactly the same time after COVID diagnosis is not explained<br>• High proportion of non-maximal studies
Brown, et al,[47] 2022	**Moderate**<br>Only hospitalized	**Moderate**<br>No information provided	**Low**<br>Novel CPET-CMR protocol	**Moderate**<br>Use of self-reported exercise capacity may not reflect LC	**Low**<br>Use of restriction/ exclusion	**Moderate**<br>No adjusted models, but well-matched	• Only included hospitalized individuals<br>• Matched on key confounders, but no adjusted models
Durstenfeld, et al,[28] 2022	**Moderate**<br>Mostly non-hospitalized convenience sample	**Moderate**<br>Only 39/120 (33%) completed CPET although differences appear minimal	**Low**<br>Cycle ergometer targeting 10 minute test, few stopped early, interpretation well-described	**Low**<br>Defined as new symptoms consistent with WHO; sensitivity analyses performed	**Low**<br>Did not assess pre-COVID fitness	**Low**<br>Adjusted models with likely confounders	• Selection Bias<br>• Attrition<br>• Confounding by pre-COVID fitness

© 2022 Durstenfeld MS et al. *JAMA Network Open*.

First Author, Year	Study Participation	Study Attrition	CPET Protocol, Execution & Interpretation	Assessment of LC Symptoms	Confounding	Statistical Analysis & Reporting	Key Threats to Validity
Ladlow et al,[34] 2022	**Low**<br>Includes active duty military with appropriate controls	**Low**<br>113/150 (75)	**Low**<br>Cycle ergometer targeting 10 minute test	**Moderate**<br>Presence of one or more symptoms may be overly sensitive and not specific	**High**<br>Stratification by severity of illness; did not account for BMI differences	**High**<br>No adjusted models	• Even though all participants had prior exercise testing, these results are not reported or used to adjust for pre-COVID fitness<br>• No adjustment for confounders (ie BMI)
Margalit et al,[40] 2022	**Moderate**<br>Mostly non-hospitalized individuals attending COVID recovery clinic	**Moderate**<br>Included 113/462 (24%) of those randomly sampled	**Moderate**<br>Treadmill<br>Low average RER<br>No individual interpretation of studies	**Low**<br>Well described assessment of LC fatigue, included sensitivity analyses	**Moderate**<br>Extensive measurement of possible confounders, but unclear if incorporate into models	**Moderate**<br>No description of variables included in models	• Selection bias: Most of the randomly sampled individuals from within the LC clinic were ineligible or did not agree to participate<br>• Low average RER suggests submaximal CPET<br>• Lack of description of statistical models
Schaeffer et al,[48,69] 2021	**Moderate**<br>Only hospitalized	**Low**<br>49/91 (54%) completed CPET	**Low**<br>15 W/min cycle ergometer	**Low**<br>Binary fatigue variable does not account for pre-COVID fatigue	**Moderate**<br>Excluded comorbidities, but higher BMI in fatigue group	**High**<br>No adjusted models, but sensitivity analysis with % predicted	• Selection bias (only hospitalized)<br>• Did not account for confounders in analysis, but reported both absolute and percent predicted
Skjørten et al,[36] 2021	**Moderate**<br>Only hospitalized	**Low**<br>156/236 (66%) completed “adequate” CPET and not excluded	**Moderate**<br>Treadmill, modified Bruce<br>Low average RER<br>Wasserman algorithm	**Moderate**<br>Use mMRC dyspnea scale 0 vs 1-4	**High**<br>Excluded comorbidities, but higher BMI in dyspnea group	**High**<br>Only adjust for age & sex	• Selection bias (only hospitalized)<br>• Low average RER suggests submaximal CPET<br>• Adjusted models only adjust for age and sex
Szekely et al,[32] 2021	**Moderate**<br>Emergency department during acute infection and attended LC Clinic	**Low**<br>71/165 (43%); flowchart, but differences between those	**Low**<br>Semi-supine cycle ergometer targeting 10 minute test	**Moderate**<br>No description of how dyspnea & fatigue were assessed	**Moderate**<br>Forced age & sex into models, but did not include BMI, severity, and	**High**<br>Stepwise multivariable analysis left out confounders and	• Selection bias from only including those who sought care acutely and followed up in LC Clinic<br>• Data-driven analysis left out important confounders (BMI, for example) and adjusted for likely

© 2022 Durstenfeld MS et al. *JAMA Network Open*.

		assessed and not			other confounders	adjusted for mediators	mediators (stroke volume, TAPSE, HR, A-Vo_2 difference)

© 2022 Durstenfeld MS et al. *JAMA Network Open*.

eTable 3. Quality Assessment and Potential Threats to Validity Among Studies Included in Assessment of Limitations of Exercise Capacity

First Author, Year	Reduced Def.	Reduced Peak VO_2, n (%)	Study Participation	Study Attrition	CPET Protocol, Execution & Interpretation	LC Symptoms	Confound-ing	Statistical Analysis & Reporting	Key Threats to Validity Pertinent to Classification
Abdallah/ Schaeffer et al,[48,69] 2021	<85%	41/63 (65)	**Moderate** Prospective cohort of only hospitalized	**Low** 49/91 (54) completed CPET	**Low** Cycle ergometer fixed protocol 15 W/min step	**Low** Binary fatigue variable does not account for pre-COVID fatigue	**Moderate** Excluded comorbidities, but higher BMI in fatigue group	**High** No adjusted models, but sensitivity analysis with % predicted	• Selection bias<br>• Confounding (pre-existing medical comorbidities, beta blockers)<br>• CPET interpretation not described
Alba et al,[45] 2021	<80%	6/18 (33)	**High** Retrospective cohort referred for CPET from LC Clinic	**High** Not reported	**Low** Upright cycle ergometer, excluded low RER	**Moderate** mMRC dyspnea scale	**High** Not addressed	**High** No adjusted models	• Small samples size<br>• Selection bias<br>• High proportion with preexisting cardiopulmonary disease
Ambrosino et al,[59] 2022	<20 ml/kg/ min	28/36 (78)	**High** Pulmonary rehab after severe COVID-19, mostly on long-term oxygen	**Moderate** 36/112 (32)	**Low** Cycle ergometer, no low RER (or excluded)	**N/A**	**Low** Adjusted	**Low** Adjusted models include most confounders	• Selection bias: all severe COVID mostly still on oxygen<br>• Unclear time after infection<br>• Lack of interpretation of individual studies
Aparisi, et al,[51] 2021	NR		**Moderate** Prospective cohort mostly hospitalized	**Moderate** 53/522 (10) of hospitalized and few non-hospitalized	**Moderate** Treadmill ramp Low average RER	**Moderate** Used standardized non-COVID tools for dyspnea	**High** Not addressed	**High** No adjusted models	• Selection Bias<br>• Low average RER suggests submaximal CPET<br>• Confounding<br>• Lack of interpretation of individual studies
Barbagelata et	<85%	39/112 (35)	**High** Retrospective EHR-	**Moderate** No	**High** Treadmill with individualized	**High** Dyspnea or fatigue >45	**Moderate** Adjust for gender,	**Moderate**	• High proportion of non-maximal studies (RER<1.1 for 47% of

© 2022 Durstenfeld MS et al. *JAMA Network Open*.

al,[41], 2021			based study without explanation for why individuals without LC underwent CPET	information provided	Bruce/ modified Bruce High proportion low RER studies	days after symptom onset but ascertained through chart review	cardiovascular history, and use of beta blockers	Data-driven variable selection	studies and 49% did not reach anaerobic threshold)<br>• High prevalence of cardiovascular disease and risk factors
First Author, Year	Reduced Def.	Reduced Peak VO_2, n (%)	Study Participation	Study Attrition	CPET Protocol, Execution & Interpretation	LC Symptoms	Confound-ing	Statistical Analysis & Reporting	• Key Threats to Validity Pertinent to Classification
Borrego Rodriguez et al,[70] 2021	<100 %	32/57 (56)	**Low** Non-hospitalized health care workers	**Moderate** Not reported	**Moderate** Details not reported	**Moderate** Dyspnea on exertion >3 months after infection	**High** Excluded structural heart disease	**High** No adjusted models	• Confounding<br>• Use of unconventional <100% cutoff<br>• Interpretation not described (abstract only)
Brown, et al,[47] 2022	Self-reported reduced exercise capacity	20/40 (50)	**Moderate** Prospective cohort of hospitalized without ICU stay, myocardial injury, or comorbidities	**Moderate** Not reported	**Low** Novel CPET-CMR protocol using supine cycle ergometer	**Moderate** Use of self-reported exercise capacity may not reflect LC	**Low** Use of restriction/ exclusion	**Moderate** No adjusted models, but well-matched	• Only included hospitalized individuals
Cassar et al,[29,71] 2021	<80%	6/31 (19)	**Moderate** Prospective cohort after COVID hospitalization	**Low** 46/58 (79) retained	**Moderate** Cycle ergometer 10W/min ramp, 26% submaximal tests	**Low** Use validated scales and longitudinal symptom assessment	**Low** Group matched controls	**Moderate** Details of adjusted analyses are not provided	• Only included hospitalized individuals<br>• Confounding
Clavario et al,[27] 2021	<85%	99/200 (50)	**Moderate** Prospective cohort after COVID	**Low** 200/225 (89)	**Low** Cycle ergometer targeting 10 minute test	**N/A**	**High** Included patients with HF,	**Moderate** Data-driven variable selection	• Only included hospitalized individuals<br>• Confounding

© 2022 Durstenfeld MS et al. *JAMA Network Open*.

			hospitalization		Independent interpretation by 2 reviewers		COPD, MI		
First Author, Year	Reduced Def.	Reduced Peak VO_2, n (%)	Study Participation	Study Attrition	CPET Protocol, Execution & Interpretation	LC Symptoms	Confound-ing	Statistical Analysis & Reporting	Key Threats to Validity Pertinent to Classification
de Boer et al,[43] 2021	<84%	16/50 (32)	**High** Retrospective case series of clinically referred CPETs for PASC	**High** Not reported	**Low** Cycle ergometer ramp	**N/A**	**Moderate** Address through stratification	**High** No adjusted models	• Focus on compromised mitochondrial function estimated from stoichiometric equations<br>• Selection bias
Debeaumont et al,[33] 2021	<85%	12/23 (52)	**High** Retrospective case series of hospitalized COVID patients referred for CPET	**High** Not reported	**Low** Cycle ergometer customized to target	**Low** Use mMRC scale for dyspnea	**High** Not addressed	**High** No adjusted models	• Only included hospitalized individuals subsequently referred for CPET
Dorelli et al,[52,53] 2021	NR	NR	**High** Prospective cohort post-hospitalization <65 years old without comorbidities	**Moderate** 28/130 (22)	**Low** Cycle ergometer targeting 8-12 minute test	**N/A**	**Moderate** Restricted patients with comorbidities including obesity	**High** Unclear why authors want to use models to predict ventilatory inefficiency and no justification for variables considered	• Primary comparison is between those with and without exercise ventilatory inefficiency<br>• Lack of interpretation of individual studies
Durstenfeld, et al, [28] 2022	<85%	15/39 (38)	**Moderate** Prospective cohort mostly non-hospitalized	**Moderate** Only 39/120 (33%) completed	**Low** Cycle ergometer targeting 10 minute test,	**Low** Defined as new symptoms consistent	**Low** Did not assess pre-	**Low** Adjusted models with likely confounders	• Selection Bias<br>• Confounding by pre-COVID fitness

© 2022 Durstenfeld MS et al. *JAMA Network Open*.

			convenience sample without cardiovascular disease	CPET although differences appear minimal	few studies stopped early, interpretation well-described	with WHO; sensitivity analyses performed	COVID fitness		
First Author, Year	Reduced Def.	Reduced Peak VO_2, n (%)	Study Participation	Study Attrition	CPET Protocol, Execution & Interpretation	LC Symptoms	Confound-ing	Statistical Analysis & Reporting	Key Threats to Validity Pertinent to Classification
Evers et al,[58] 2022,	<100 % predicted work	11/30 (37)	**High** Retrospective case series of patients referred for post-COVID exercise limitation or dyspnea	NR 16/30 (53) underwent repeat CPET	**Low** Cycle ergometer targeting <12 minute test	**Low** mMRC dyspnea scale	**High** Not addressed	**High** No adjusted models	• Selection bias
Frésard, et al,[54] 2022	>84% NR		**High** Retrospective cohort of clinical CPETs referred for LC and persistent dyspnea	**High** Not reported	**Low** Cycle ergometer target 10 minute test	**Moderate** Use validated scales from non-COVID settings	**High** Not addressed	**High** No adjusted models	• Primary comparison is dysfunctional breathing (mostly mild-moderate COVID) compared to ventilatory limitation (mostly severe COVID)
Godinho et al,[72] 2021	NR	5/10 (50)	**High** Case series of non-hospitalized patients with persistent exercise limitations referred for clinical CPET	**High** Not reported	**High** No information provided	**N/A**	**High** Not addressed	**High** No adjusted models	• Very small case series with lack of adequate details to assess quality from abstract and no preprint or manuscript available

© 2022 Durstenfeld MS et al. *JAMA Network Open*.

Jahn et al,[73] 2021	<83%	19/35 (54%)	**Moderate** Case series of patients with severe COVID pneumonitis attending post-hospitalization on pulmonary rehab	**Low** 35/44 (80)	**Low** Semi-recumbent cycle ergometer, interpretation described	**Moderate** Use validated scales from non-COVID settings, but 60% missing	**High** Not addressed, did not exclude prior disease	**High** No adjusted models	• Selection bias (severe COVID only)<br>• Confounding
First Author, Year	Reduced Def.	Reduced Peak VO_2, n (%)	Study Participation	Study Attrition	CPET Protocol, Execution & Interpretation	LC Symptoms	Confound-ing	Statistical Analysis & Reporting	Key Threats to Validity Pertinent to Classification
Johnsen et al,[74] 2021	<84%	16/31 (52)	**High** Case-series of post COVID clinic referrals for CPET to evaluate symptoms	**High** 34/117 (29) + 23 outpatients, but unclear which 31 were included for CPET	**High** Minimal information provided	**Moderate** Detailed clinical phenotyping but not described for those who underwent CPET	**High** Not addressed for CPET	**High** Adjusted models for symptom variables for age and sex, but not for CPET	• Focus of paper is clinically phenotyping LC; does not provide adequate detail about CPET
Kersten et al,[57] 2021	NR	17/35 (55)	**High** Case-series of post COVID clinic referrals for CPET if initial testing abnormal or not revealing	**High** 36/231 (16) targeted for symptomatic	**High** Treadmill ramp, interpretation strategy not described and only summary CPET findings reported	**Moderate** Minimal information provided	**High** Not addressed	**High** Descriptive only	• Selection bias<br>• High attrition<br>• Those who underwent CPET are not well described<br>• CPET data are not reported, only categorization of reason for limitation
Ladlow et al,[34] 2022	<85%	4/61 (7)	**Low** Prospective cohort of active-duty military	**Low** 113/150 (75)	**Low** Cycle ergometer targeting 10 minute test	**Moderate** Presence of one or more symptoms	**High** Stratification by severity of illness; did	**High** No adjusted models	• Selection bias: active military personnel<br>• No description of interpretation

© 2022 Durstenfeld MS et al. *JAMA Network Open*.

			personnel with appropriate controls			may be overly sensitive and not specific	not account for sex, age, BMI		
Liu et al,[56] 2021	NR	NR	**Moderate** Prospective post-hospitalization cohort	**High** Not reported	**Moderate** Treadmill, interpretation not described or reported	**N/A**	**High** Not addressed	**High** Adjusted models to predict pulmonary fibrosis at 7 months, but model development strategy not described	• Focus of paper is pulmonary fibrosis at 7 months; does not provide adequate detail about CPET findings or interpretation or classify participants by symptoms
First Author, Year	Reduced Def.	Reduced Peak VO_2, n (%)	Study Participation	Study Attrition	CPET Protocol, Execution & Interpretation	LC Symptoms	Confound-ing	Statistical Analysis & Reporting	Key Threats to Validity Pertinent to Classification
Mancini et al,[46] 2021	<80%	24/41 (59)	**High** Case-series of LC clinic referrals for CPET for dyspnea with normal cardiopulmonary testing	**High** Not reported	**Low** Cycle ergometer 25 W/3 minute step, subset with invasive ("hemodynamic") CPET; classification well described	**Low** Interview for ME/CFS symptoms	**Moderate** Used % predicted; excluded known cardiopulmonary disease; high proportion on beta blockers, not held; other confounders not addressed	**High** No adjusted models	• Selection bias (LC Clinic referrals)<br>• Confounding (ie beta blocker use)
Margalit et	NR		**Moderate** Nested case-control	**Moderate** 113/462 (24)	**Moderate** Treadmill with low average	**Low** Well described	**Low** Extensive measurem	**Moderate** No description	• Selection bias: All sampled individuals were from LC Clinic; most of

© 2022 Durstenfeld MS et al. *JAMA Network Open*.

al,[40] 2022			study within COVID recovery cohort of mostly non-hospitalized individuals attending COVID recovery clinic	randomly sampled	RER; no individual interpretation	assessment of LC fatigue, included sensitivity analyses	ent of possible confounders, and well-balanced groups	of variables included in models	the randomly sampled individuals from within the LC clinic were ineligible or did not agree to participate<br>• Low average RER suggests submaximal CPET
First Author, Year	*Reduced Def.*	*Reduced Peak VO_2, n (%)*	*Study Participation*	*Study Attrition*	*CPET Protocol, Execution & Interpretation*	*LC Symptoms*	*Confound-ing*	*Statistical Analysis & Reporting*	*Key Threats to Validity Pertinent to Classification*
Mohr et al,[44] 2021	<85%	8/10 (80)	**High** Retrospective case-series of post COVID clinic referrals for CPET for dyspnea	**High** 10/42 (24)	**High** CPET methods and interpretation not described	**High** Not described	**High** Not addressed	**High** Descriptive only; no adjusted models	• Small sample size<br>• Selection bias<br>• Inadequate description of CPET methods and interpretation<br>• Heterogeneity within sample without addressing likely confounders
Motiejunaite et al,[50] 2021	<85%	86/114 (75)	**High** Prospective cohort but target population and recruitment not well-described	**High** Not reported	**Moderate** Cycle ergometer, interpretation well described	**High** Symptom assessment not described	**High** Not addressed	**High** Compared reduced to preserved diffusing capacity; no adjusted models	• Analytic focus is comparing those with reduced vs. preserved diffusing capacity
Moulson et al,[61] 2022	<80%	3/21 (14)	**High** Case-series of young athletes referred for cardiopulmonary	**Moderate** 13/21 (62) retained	**Moderate** Treadmill or cycle ergometer, protocols & interpretation well described	**Low** Interview for symptoms	**Low** Only included young athletes without comorbidities and	**High** Descriptive only; no adjusted models for symptoms	• Selection bias: only included symptomatic athletes<br>• Attrition for longitudinal CPET

© 2022 Durstenfeld MS et al. *JAMA Network Open*.

			symptoms after COVID				compared to similar reference group of athletes		
Parkes et al,[75] 2021	<85%	10/12 (83)	**High**<br>Retrospective cohort of clinical CPETs	**High**<br>12/600 (2)	**High**<br>Not described; sub-max tests are hinted at	**High**<br>Not described	**High**<br>Not addressed	**High**<br>No adjusted models	• Small sample size<br>• Selection bias<br>• Inadequate description of CPET methods and interpretation
Pleguezuelos, et al,[60] 2021	NR		**High**<br>Case series of survivors of ARDS from COVID pneumonia requiring mechanical ventilation & tracheostomy	**High**<br>Not reported	**Low**<br>Cycle ergometer targeting 6-12 minute test	**High**<br>Not described	**High**<br>Not addressed, but compared to multiple reference groups	**High**<br>No adjusted models	• Focus is comparing mechanical efficiency among those with severe COVID to those with COPD, ischemic heart disease, and healthy controls<br>• Selection bias: only included patients requiring prolonged ICU care<br>• Confounding
First Author, Year	Reduced Def.	Reduced Peak VO_2, n (%)	Study Participation	Study Attrition	CPET Protocol, Execution & Interpretation	LC Symptoms	Confound-ing	Statistical Analysis & Reporting	Key Threats to Validity Pertinent to Classification
Ribeiro Baptista, et al,[39] 2022	<80%	37/105 (35)	**Moderate**<br>Prospective cohort of severe COVID requiring hospitalization >7 days and oxygen (43% ICU)	**Moderate**<br>105/220 (48)	**Moderate**<br>Cycle ergometer 10-20 W/min; interpretation not described	**Moderate**<br>mMRC dyspnea scale	**High**<br>Not addressed	**Moderate**<br>Stepwise backward selection for models to assess associations with reduced VO_2	• Selection bias: only included patients with severe COVID<br>• Confounding
Rinaldo, et al[38,76] 2021	<85%	41/75 (55)	**Moderate**<br>Prospective cohort post-	**High**<br>Not reported	**Moderate**<br>Cycle ergometer with	**Moderate**<br>mMRC dyspnea scale	**High**<br>Not addressed	**High**<br>No adjusted models	• Selection bias<br>• Overly simplistic interpretation of abnormal studies

© 2022 Durstenfeld MS et al. *JAMA Network Open*.

			hospitalization		individualized protocol, but classification by breathing reserve and heart rate reserve is overly simplistic				• Confounding not addressed
Singh et al,[42] 2021	<80%	NR	**High** Prospective cohort of patients referred for CPET from LC Clinic for unexplained exercise intolerance with negative initial workup	**High** Not reported	**Moderate** Invasive CPET including pulmonary artery and radial artery lines with cycle ergometer with individualized protocol, but tests terminated at RER>1.1 or HR>85% predicted	**N/A**	**High** Matching by age and sex but not other potential confounders (ie BMI higher in COVID than controls)	**High** No adjusted models	• Selection bias<br>• Termination of exercise based on submaximal criteria
First Author, Year	Reduced Def.	Reduced Peak VO_2, n (%)	Study Participation	Study Attrition	CPET Protocol, Execution & Interpretation	LC Symptoms	Confound-ing	Statistical Analysis & Reporting	Key Threats to Validity Pertinent to Classification
Skjørten et al,[36] 2021,	<80%	49/156 (31)	**Moderate** Multicenter prospective cohort only hospitalized	**Low** 156/236 (66%) completed "adequate" CPET and were not excluded for	**Moderate** Treadmill, modified Bruce Low average RER Wasserman algorithm	**Moderate** Use mMRC dyspnea scale 0 vs 1-4	**High** Excluded comorbidities, but higher BMI in dyspnea group	**High** Only adjust for age & sex	• Low average RER suggests submaximal CPET

© 2022 Durstenfeld MS et al. *JAMA Network Open*.

				comorbidity					
Szekely et al,[32] 2021	<85%	49/71 (69)	**Moderate**<br>Prospective cohort of individuals who went to emergency department for acute COVID-19 and attended LC Clinic	**Low**<br>71/165 (43%) with clear flowchart, but with some differences between those assessed and those not assessed	**Low**<br>Semi-supine cycle ergometer targeting 10 minute test	**Moderate**<br>No description of how dyspnea & fatigue were assessed	**Moderate**<br>Forced age & sex into models, but did not include BMI, severity, and other confounders	**High**<br>Stepwise multivariable analysis left out confounders and adjusted for mediators	• Selection bias<br>• Interpretation of individual studies not described
First Author, Year	Reduced Def.	Reduced Peak VO_2, n (%)	Study Participation	Study Attrition	CPET Protocol, Execution & Interpretation	LC Symptoms	Confound-ing	Statistical Analysis & Reporting	Key Threats to Validity Pertinent to Classification
Vannini et a;,[77] 2021	<80%	19/41 (46)	**Moderate**<br>Prospective cohort post-hospitalization	**High**<br>Not reported	**Moderate**<br>Cycle ergometer 10W/min ramp; interpretation not described	**N/A**	**High**<br>Stratification by severity of acute COVID; other confounders not addressed	**High**<br>No adjusted models	• Selection Bias<br>• Confounding not adequately addressed<br>• CPET interpretation not described
von Gruenewaldt et al,[55] 2022	<80%	2/20 (10)	**High**<br>Retrospective cohort of clinical CPETs	**High**<br>Not reported	**Moderate**<br>Cycle ergometer 10 or 20W/min ramp targeting 10 minute test; interpretation focused on dysfunctional breathing	**High**<br>Symptoms assessed through records; participants without PCR verified diagnosis	**High**<br>Not addressed	**High**<br>No adjusted models	• Small sample size<br>• A priori focus is abnormal breathing pattern<br>• Unclear if other interpretations were considered

© 2022 Durstenfeld MS et al. *JAMA Network Open*.

Vonbank et al,[37] 2021	NR		**Low** Prospective cohort including full spectrum of acute SARS-CoV-2 infection	**High** Not reported	**Moderate** Cycle ergometer targeting 8-12 minute test; no interpretation of individual studies	**N/A**	**Moderate** Addressed through adjusted model, but not all included	**High** Stepwise multivariable analysis left out confounders and adjusted for mediators	• Focus is comparing exercise capacity by severity of acute illness to healthy controls<br>• Interpretation not described

© 2022 Durstenfeld MS et al. *JAMA Network Open*.

eMethods. Study Protocol

The full, pre-registered Protocol is available at
https://www.crd.york.ac.uk/prospero/display_record.php?ID=CRD42021299842.
This review was conducted according to the PRISMA (Preferred Reporting Items for Systematic Reviews and Meta-Analysis)[25] guidelines and was registered prospectively on PROSPERO prior to beginning the search.

Condition being studied: post-acute sequelae of COVID-19, also known as Long COVID, which according to the WHO definition is >3 months after acute infection with SARS-CoV-2.

Inclusion criteria: all studies of adults with confirmed COVID-19 at least 3 months after onset that include cardiopulmonary exercise testing with measurement of peak VO_2 published since 2020 will be included. Baseline cardiopulmonary exercise testing from interventional or randomized controlled trials will also be included if they meet the other inclusion criteria.

Exclusion criteria: studies of children, studies of conditions other than COVID-19/SARS-CoV-2, studies in the acute or early post-acute phase (<3 months after infection), review articles, case reports.

Intervention/exposure: Cardiopulmonary exercise testing, which includes measurement of metabolic gases with either treadmill or cycle ergometer exercise.

Participants/population: We are interested in all adults with COVID without respect to hospitalization status or severity of acute illness.

Inclusion/Exclusion criteria: adults with confirmed COVID-19 at least 3 months after onset that include cardiopulmonary exercise testing with measurement of peak VO_2 will be included. We excluded studies of children, studies of conditions other than COVID-19/SARS-CoV-2, studies in the acute phase (<3 months after infection).

Comparators/control: We will include case series without controls, as well as studies with healthy controls, control participants with unexplained dyspnea, or that compare those who have fully recovered from COVID compared to those reporting ongoing symptoms.

Types of studies to be included: We will include observational studies including case series, cross-sectional studies, case-control studies, and cohort studies. We will also include randomized trials of interventions, in which case we will use baseline CPET data. We will exclude case reports and review articles.

Context: We will include studies that include the full spectrum of COVID-19; specifically, we will not restrict to only studies of those requiring ICU or hospitalization during acute infection.

Main Outcomes: The primary outcome will be peak VO_2 (in ml/kg/min and % predicted). If meta-analysis is possible, studies that do not include this measure will be excluded from meta-analysis. We will report the difference in peak VO_2 between those with and without COVID and among those with COVID between those with and without post-acute sequelae.

Additional outcomes: Additional outcomes will include the proportion with exercise limitation <80 or 85% of predicted (different studies use different cutoffs), difference in exercise capacity between those with and without cardiopulmonary symptoms (absolute and relative difference with 95% confidence intervals and p value), common features among those with limitations (i.e., reduced oxygen pulse pressure, reduced chronotropic response). We will likely report these effect measures in odds-ratios as we expect that many of the studies may be case-control studies.

Search Strategy & Information Sources: A comprehensive, electronic search strategy will be used to identify studies published since 2020 and indexed in PubMed, EMBASE, and Web of Science by a research librarian (PT) with extensive experience in systematic reviews. Unpublished abstracts from conference proceedings and indexed preprints will be included as part of our gray literature search. We will also review references from studies selected for data extraction. The search strategy will include terms and synonyms for the following: (COVID or SARS-CoV-

© 2022 Durstenfeld MS et al. *JAMA Network Open*.

2) AND ("cardiopulmonary exercise test*" OR (CPET or CPX or CPEX) OR exercise capacity OR VO2 OR anaerobic threshold). Searches will be tailored to each database depending on indexing terminology. Searches were conducted on December 20, 2021, and rerun prior to the final analysis on May 24, 2022; pre-prints were searched through June 9, 2022. Abstracts were reviewed for inclusion by two independent reviewers (MSD & KS); if there is disagreement after consensus discussion, a third reviewer will be consulted. All data extraction was done independently, in duplicate, using REDCap for data entry.

Gray literature plan: see search strategy for details; we will review conference abstracts, pre-prints, and references from studies that meet the inclusion criteria.

Data Extraction (Selection & Coding): Data including authors, title, date of study, location of study, sample size (including total with COVID, total with Cardiopulmonary Long COVID, and COVID-negative controls, if included), median time since acute infection and interquartile range, inclusion criteria (with particular attention to inclusion of hospitalized/ICU/ambulatory during acute illness and those with specific comorbidities or populations of interest), comparator group, exercise modality (treadmill or cycle ergometer), peak VO2 (in ml/kg/min and % predicted), proportion with exercise limitation <85% of predicted, difference in exercise capacity between those with and without cardiopulmonary symptoms (absolute and relative difference with 95% confidence intervals and p value), common features among those with limitations (i.e., reduced oxygen pulse pressure, reduced chronotropic response). If available, other cardiopulmonary parameters will be recorded including echocardiographic, pulmonary function tests, chest computed tomography, and cardiac magnetic resonance imaging.

Data Management: Studies identified through the searches will be managed using Covidence. Data extracted will be recorded using REDCap.

Quality Assessment: We will use Cochrane's Quality in Prognostic Studies (QUIPS) tool to assess for bias of included studies. We will assess study populations (especially choice of control groups), study attrition for non-cross-sectional studies, peak VO_2 assessment quality, outcome measurement, study confounding, and statistical analysis and reporting. We will use Cochrane's Quality in Prognostic Studies (QUIPS) tool to assess for bias of included studies.

Data synthesis: Overall findings of each study will be summarized in a table. If possible, a meta-analysis will be performed to compare the peak VO2 among those with and without COVID. An odds ratio of having reduced exercise capacity may also be estimated if possible. Heterogeneity will be assessed using I^2. The primary subgroup we plan to investigate is to compare peak VO2 (and the other explanatory variables for reduced exercise capacity) among those with and without PASC/Long COVID. If possible, we may also compare those with severe acute infection requiring hospitalization and/or ICU care with those who were asymptomatic or had mild acute infection. Lastly, we may compare the early post-acute period (3-6 months), medium term (6-12 months), and long term (>12 months). Analyses will be performed using STATA version 17.

Analysis of subgroups: The primary subgroup we plan to investigate is to compare peak VO2 (and the other explanatory variables for reduced exercise capacity) among those with and without PASC/Long COVID. If possible, we may also compare those with severe acute infection requiring hospitalization and/or ICU care with those who were asymptomatic or had mild acute infection. Lastly, we may compare the early post-acute period (3-6 months), medium term (6-12 months), and long term (>12 months).

Risk of Bias/Quality Assessment: Risk of bias will be assessed at both the study and the outcome level for each included study. Publication bias will be assessed using a Funnel Plot. The strength of the body of evidence will be assessed using the GRADE (Grading of Recommendations, Assessment, Development, and Evaluations) framework.

© 2022 Durstenfeld MS et al. *JAMA Network Open*.

eAppendix. Study Findings and Quality Form

Record ID	______________________________
Extractor	◯ Matt<br>◯ Kevin
First author's last name	______________________________
all authors	______________________________
Title	______________________________
year	◯ 2020<br>◯ 2021<br>◯ 2022
Journal	______________________________
Type of Publication	◯ Full Manuscript<br>◯ Research Letter<br>◯ Abstract/Conference Proceedings<br>◯ Non-English Full Manuscript<br>◯ Review<br>◯ Other (comments)
Duplicate with (enter other 1st author/Journal)	______________________________
study type	◯ prospective cohort (research CPETs)<br>◯ retrospective cohort<br>◯ case-control<br>◯ case-series (ie patients referred for clinically ordered CPETs)<br>◯ other
study type comments	______________________________

© 2022 Durstenfeld MS et al. *JAMA Network Open*.

Study Location (City/State/Country/Region)	________________________
Start date of study	________________________
End date of study	________________________
total sample size who underwent CPET	________________________
Median time since acute infection (days)<br>-if reported in months multiply by 30<br>-if reported in weeks multiply by 7	________________________
Interquartile Range time since acute infection (days)<br>-if reported in months multiply by 30<br>-if reported in weeks multiply by 7	________________________
If median/IQR time since infection not reported,<br>then put mean and standard deviation here	________________________
**Inclusion Criteria**	
Inclusion Criteriano testing required<br>How was COVID diagnosed?PCR confirmed acute infection	○<br>○<br>○<br>○ antibody testing<br>other
Inclusion Criteria:<br>age	________________________
Mean or median age	________________________
age standard deviation	________________________
number female	________________________

© 2022 Durstenfeld MS et al. *JAMA Network Open*.

female %

____

Inclusion
hospitalization

○ Included patients irrespective of hospitalization
○ Included only patients hospitalized for acute disease
○ INcluded only
○ Other (note in comments)

Inclusion
ICU

○ Included patients irrespective of ICU admission
○ Included only patients admitted to ICU for acute disease
○ Other (note in comments)

Inclusion Criteria
Are only athletes

○ No being an athlete is not required
○ Yes only athletes

Inclusion
Comorbidities/Special

○ No specific comorbidities required for entry
○ Specific comorbidities required (ie heart failure)

Inclusion
Comorbidities required for inclusion

Primary comparison

____

Sample Size of control group WITHOUT COVID

____

Peak VO2 (ml/kg/min) among controls WITHOUT COVID

____

Peak VO2 (% predicted) among controls WITHOUT COVID

____

Among those without COVID, proportion with exercise limitation (0 to 1.00)

____

Sample Size who had COVID

____

© 2022 Durstenfeld MS et al. *JAMA Network Open*.

Peak VO2 (ml/kg/min) among all WITH COVID	___________________________________
Peak VO2 (% predicted) among all WITH COVID	___________________________________

Among those WITH COVID, proportion with exercise limitation (0 to 1.00)	___________________________________
Sample Size with COVID but without PASC/Long COVID	___________________________________
Peak VO2 (ml/kg/min) WITH COVID but without PASC	___________________________________
Peak VO2 (% predicted) WITH COVID but WITHOUT PASC	___________________________________
Sample Size with PASC/Long COVID	___________________________________
Peak VO2 (ml/kg/min) among those WITH PASC/Long COVID	___________________________________
Peak VO2 (% predicted) among those WITH PASC/Long COVID	___________________________________
Number with reduced exercise capacity	___________________________________
Among those WITH PASC/LONG COVID, proportion with exercise limitation (0 to 1.00)	___________________________________

Definition of Exercise Limitation

© 2022 Durstenfeld MS et al. *JAMA Network Open*.

Exericse modality cycle ergometer	◯ ◯ ◯ treadmill other (list in comments) ______________________
Difference in peak VO2 (ml/kg/min) Cardiopulmonary PASC vs no PASC	______________________
Confidence interval of Difference in peak VO2 (ml/kg/min) Cardiopulmonary PASC vs no PASC	______________________
Difference in peak VO2 (% predicted) Cardiopulmonary PASC vs no PASC	______________________
Confidence interval of Difference in peak VO2 (% predicted) Cardiopulmonary PASC vs no PASC	______________________
Relative exercise capacity (RR) among those with PASC vs no PASC	______________________
Confidence interval of relative exercise capacity Cardiopulmonary PASC vs no PASC	______________________
Primary etiology of reduced exercise capacity in PASCNo	◯ ◯ ◯ ◯ ◯ ◯ ◯ ◯ primary etiology Deconditioning Ventilatory Limitation Cardiac Limitation Chronotropic Multifactorial Other Peripheral
Proportion with PASC with deconditioning	______________________
Proportion with PASC with ventilatory limitation	______________________
Proportion with PASC with cardiac limitation	______________________

© 2022 Durstenfeld MS et al. *JAMA Network Open*.

Proportion with PASC with peripheral limitation (oxygen extraction/utilization) ________________________________

Proportion with PASC with chronotropic incompetence ________________________________

Other reason for limitation reported ________________________________

Proportion with PASC with Other Limitation ________________________________

Other objective data available

- ☐ None
- ☐ Rest echo
- ☐ Stress echo
- ☐ Chest CT
- ☐ CMR
- ☐ Inflammatory markers
- ☐ cardiac biomarkers
- ☐ right heart cath
- ☐ PFTs
- ☐ 1st pass ventriculography
- ☐ lactate/arterial blood gas

Primary analytic comparison reported ____________________________

Study Quality Assessment
**1. Study Participation**
**Goal: To judge the risk of selection bias (likelihood that relationship between PF and outcome is different for participants and eligible non-participants).**

Source of target population<br>The source population or population of interest is adequately described for key characteristics (age, sex, hosp/ICU, time since COVID, special populations comorbidities, precovid fitness)	◯ No<br>◯ Partial<br>◯ Yes<br>◯ Unsure
Method used to identify population:<br>The sampling frame and recruitment are adequately described, including methods to identify the sample sufficient to limit potential bias (number and type used, e.g., referral patterns in health care)	◯ No<br>◯ Partial<br>◯ Yes<br>◯ Unsure
Period of recruitment is adequately described	◯ No<br>◯ Partial<br>◯ Yes<br>◯ Unsure
Place of recruitment (setting and geographic location) are adequately described	◯ No<br>◯ Partial<br>◯ Yes<br>◯ Unsure
Inclusion and exclusion criteria are adequately described (e.g., including explicit diagnostic criteria or "zero time" description).	◯ No<br>◯ Partial<br>◯ Yes<br>◯ Unsure
Adequate study participation	◯ No<br>◯ Partial<br>◯ Yes<br>◯ Unsure
The baseline study sample (i.e., individuals entering the study) is adequately described for key characteristics:<br>(age, sex, hosp/ICU, time since COVID, special populations comorbidities, precovid fitness).	◯ No<br>◯ Partial<br>◯ Yes<br>◯ Unsure

Overall comments on study populations

____

Overall comments on control groups? ____

Summary Study Participation<br>The study sample represents the population of interest on key characteristics, sufficient to limit potential bias of the observed relationship between PF and outcome	◯ Low risk<br>◯ Moderate risk<br>◯ High risk

2. Study Attrition

Goal: To judge the risk of attrition bias (likelihood that relationship between PF and outcome are different for completing and non-completing participants).

© 2022 Durstenfeld MS et al. *JAMA Network Open*.

Proportion of baseline sample available for analysis Response rate (i.e., proportion of study sample completing the study and providing outcome data) is adequate.	○ No<br>○ Partial<br>○ Yes<br>○ Unsure
number eligible	________________________________
number included	____________________________________
proportion retained	________________________________
Attempts to collect information on participants who dropped out of the study are described.	○ No<br>○ Partial<br>○ Yes<br>○ Unsure
Reasons for loss to follow-up are provided.	○ No<br>○ Partial<br>○ Yes<br>○ Unsure
Participants lost to follow-up are adequately described for key characteristics (hosp, symptoms, etc)	○ No<br>○ Partial<br>○ Yes<br>○ Unsure
There are no important differences between key characteristics (age, sex, time since COVID, severity of acute illness, comorbidities, persistent symptoms) and outcomes in participants who completed the study and those who did not.	○ No<br>○ Partial<br>○ Yes<br>○ Unsure
Study Attrition Summary<br>Loss to follow-up (from baseline sample to study population analyzed) is not associated with key characteristics (i.e., the study data adequately represent the sample) sufficient to limit potential bias to the observed relationship between PF and outcome.	○ Low Risk<br>○ Moderate Risk<br>○ High Risk
Overall comments on Study Attrition	____________________________________

© 2022 Durstenfeld MS et al. *JAMA Network Open*.

**3. Prognostic Factor Measurement**<br>**Goal: To judge the risk of measurement bias related to how PF was measured (differential measurement of PF related to the level of outcome).**	
Definition of the PF (CPET)<br>A clear definition or description of CPET is provided (e.g., including exercise modality & protocol,<br>stopping criteria, assessment of submaximal tests (ie RER, Borg, HR, double product), and clear specification of the method of measurement and classification of limitations)	◯ No<br>◯ Partial<br>◯ Yes<br>◯ Unsure
Valid and Reliable Measurement of PF (CPET)<br>Method of PF measurement is adequately valid and reliable to limit misclassification bias (e.g., may include relevant outside sources of information on measurement properties, also characteristics, such as blind measurement and limited reliance on recall)<br>Especially how tests are interpreted, how anaerobic threshold is identified	◯ No<br>◯ Partial<br>◯ Yes<br>◯ Unsure
Continuous variables are reported or appropriate cut-points (i.e., not data-dependent) are used.	◯ No<br>◯ Partial<br>◯ Yes<br>◯ Unsure
The method and setting of measurement of PF is the same for all study participants	◯ No<br>◯ Partial<br>◯ Yes<br>◯ Unsure
Adequate proportion of the study sample has complete data for PF variable.	◯ No<br>◯ Partial<br>◯ Yes<br>◯ Unsure
Appropriate methods of imputation are used for missing 'PF' data.	◯ No<br>◯ Partial<br>◯ Yes<br>◯ Unsure
PF (CPET) Measurement Summary<br><br>PF is adequately measured in study participants to sufficiently limit potential bias.<br>Overall comments on CPET quality	◯ Low risk<br>◯ Moderate risk<br>◯ High risk<br><br>______________________________

© 2022 Durstenfeld MS et al. *JAMA Network Open*.

4. Outcome Measurement	
**Goal: To judge the risk of bias related to the measurement of outcome (differential measurement of outcome related to the baseline level of PF).**	
A clear definition of outcome (PASC/Long COVID/Symptoms) is provided, including duration of follow-up and level and extent of the outcome construct	◯ No<br>◯ Partial<br>◯ Yes<br>◯ Unsure
Valid and Reliable Measurement of Outcome<br>The method of outcome measurement used is adequately valid and reliable to limit misclassification bias (e.g., may include relevant outside sources of information on measurement properties, also characteristics, such as blind measurement and confirmation of outcome with valid and reliable test).	◯ No<br>◯ Partial<br>◯ Yes<br>◯ Unsure
Method and Setting of Outcome Measurement<br>The method and setting of outcome measurement is the same for all study participants.	◯ No<br>◯ Partial<br>◯ Yes<br>◯ Unsure
Outcome (Symptoms) Measurement Summary<br><br>Outcome of interest is adequately measured in study participants to sufficiently limit potential bias.	◯ Low risk<br>◯ Moderate risk<br>◯ High risk
Overall comments on assessment of PASC/Long COVID/symptoms	____________________________________

5. Study Confounding	
**Goal: To judge the risk of bias due to confounding (i.e. the effect of PF is distorted by another factor that is related to PF and outcome).**	
Important Confounders Measured<br>All important confounders, including treatments (key variables in conceptual model: LIST), are measured.	◯ No<br>◯ Partial<br>◯ Yes<br>◯ Unsure
Definition of the confounding factor Clear definitions of the important confounders measured are provided (e.g., including dose, level, and duration of exposures).	◯ No<br>◯ Partial<br>◯ Yes<br>◯ Unsure
Valid and Reliable Measurement of Confounders<br>Measurement of all important confounders is adequately valid and reliable (e.g., may include relevant outside sources of information on measurement properties, also characteristics, such as blind measurement and limited reliance on recall).	◯ No<br>◯ Partial<br>◯ Yes<br>◯ Unsure

© 2022 Durstenfeld MS et al. *JAMA Network Open*.

Method and Setting of Confounding Measurement<br>The method and setting of confounding measurement are the same for all study participants.	○ No<br>○ Partial<br>○ Yes<br>○ Unsure
Method used for missing data<br>Appropriate methods are used if imputation is used for missing confounder data.	○ No<br>○ Partial<br>○ Yes<br>○ Unsure
Appropriate Accounting for Confounding<br>Important potential confounders are accounted for in the study design (e.g., matching for key variables, stratification, or initial assembly of comparable groups).	○ No<br>○ Partial<br>○ Yes<br>○ Unsure
Important potential confounders are accounted for in the analysis (i.e., appropriate adjustment).	○ No<br>○ Partial<br>○ Yes<br>○ Unsure
Study Confounding Summary<br><br>Important potential confounders are appropriately accounted for, limiting potential bias with respect to the relationship between PF and outcome .	○ Low risk<br>○ Moderate risk<br>○ High risk

Overall comments for counfounding

____

6. Statistical Analysis and Reporting Goal: To judge the risk of bias related to the statisticalanalysis and presentation of results

Presentation of analytical strategy<br>There is sufficient presentation of data to assess the adequacy of the analysis	○ No<br>○ Partial<br>○ Yes<br>○ Unsure
Model development strategy<br>The strategy for model building (i.e., inclusion of variables in the statistical model) is appropriate and is based on a conceptual framework or model.	○ No<br>○ Partial<br>○ Yes<br>○ Unsure
The selected statistical model is adequate for the design of the study.	○ No<br>○ Partial<br>○ Yes<br>○ Unsure
Reporting of results<br>There is no selective reporting of results.	○ No<br>○ Partial<br>○ Yes<br>○ Unsure

© 2022 Durstenfeld MS et al. *JAMA Network Open*.

Statistical Analysis and Presentation Summary

◯ Low Risk
◯ Moderate Risk
◯ High Risk

The statistical analysis is appropriate for the design of the study, limiting potential for presentation of invalid or spurious results

Overall comments regarding statistical analysis & reporting ___________________________________

© 2022 Durstenfeld MS et al. *JAMA Network Open*.

eResults. Sensitivity Analyses and GRADE Assessment

Post-hoc Sensitivity Analyses

Although peak VO_2 was higher among non-hospitalized individuals, subgroup analysis suggested that the mean difference by symptom status did not vary by the proportion hospitalized (more hospitalized: -4.7; 95%CI -6.5 to -3.0 versus fewer hospitalized: -4.6; 95%CI -7.3 to -2.0; p=0.95). Similarly, subgroup analysis comparing studies by median time after SARS-CoV-2 infection suggested that time since infection was not a major cause of heterogeneity (<6 months: -5.0; 95%CI -7.1 to -3.0; ≥6 months: -4.5; 95%CI -6.4 to -3.4; p=0.73).

Leave One Out Analysis

**Omitted study**	**Mean diff (95%CI)**
Abdallah	-4.94 (-6.58 – -3.30)
Aparisi	-4.90 (-6.62 – -3.17)
Barbagelata	-5.18 (-6.81 – -3.56)
Brown	-5.01 (-6.71 – -3.30)
Durstenfeld	-4.69 (-6.22 – -3.16)
Ladlow	-5.44 (5.93 – -3.07)
Margalit	-5.18 (-6.81 – -3.56)
Skjørten	-4.25 (-5.44 – -3.05)
Szekely	-5.18(-6.76 – -3.60)
**Overall**	**-4.87 (-6.36 – -3.39)**

<u>Summary of GRADE Assessment Discussion for Aim 1</u>

Starting for Observational Data: Low
Risk of bias: Downgrade for issues with selection bias and confounding
Imprecision: No change for precision; whether the average effect is -6 ml/kg/min or -3 ml/kg/min would not dramatically change our interpretation (although the greater estimate suggests a higher prevalence, which we were not able to estimate).
Inconsistency: Upgrade for consistency: in the subgroup analyses and leave one out analyses the effects were fairly consistent.
Indirectness: Downgrade for indirectness in measuring Long COVID symptoms.
Publication bias: Uncertain. Two studies (Clavario et al & Cassar et al) that did find a statistically significant result and therefore did not report peak VO_2 by symptom status, so it is possible that there are other negative studies that have not been published. We attempted to find these through preprints or conference abstracts in case they are having a difficult time being published.
Overall team impression: Low Certainty

<u>Summary of GRADE Assessment Discussion for Aim 2</u>

Starting for Observational Data: Low
Risk of bias: Downgrade for issues with selection bias and confounding
Imprecision: Downgrade for lack of precision especially with regards to classification of deconditioning vs muscular/peripheral issues, issues with not excluding submaximal tests.
Inconsistency: Downgrade for inconsistency; the patterns observed across different studies are not at all consistent, and some studies report negative findings that are the most common pattern observed in other studies.
Indirectness: Downgrade for indirectness in measuring “Long COVID”
Publication bias: Uncertain
Overall team impression: Very Low Certainty

© 2022 Durstenfeld MS et al. *JAMA Network Open*.

eFigure. Funnel Plot of Studies Comparing Peak $\dot{V}O_2$ Among People With and Without Symptoms

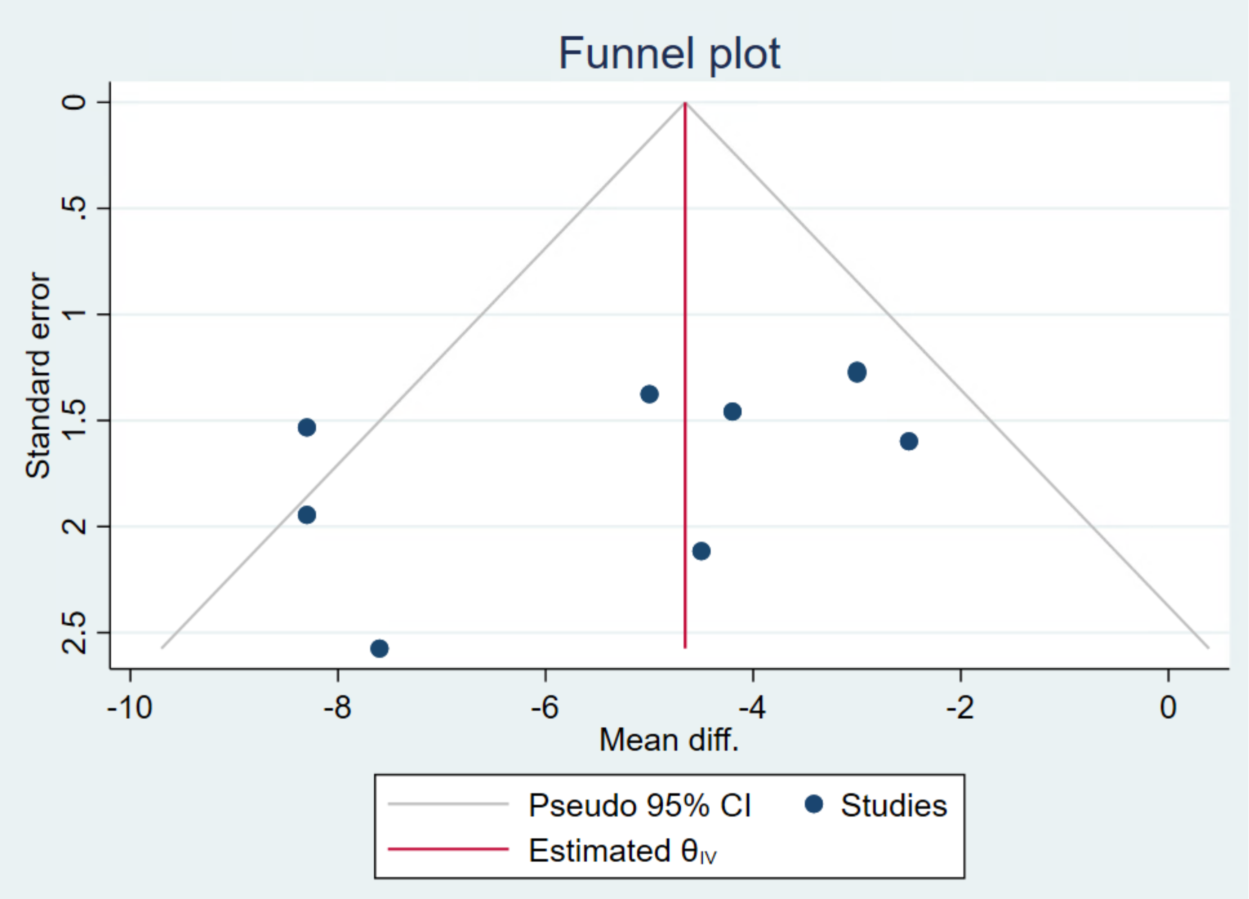

eFigure 1 Legend: Funnel plot of studies included for Aim 1 (With vs without LC Symptoms) using inverse variance.

© 2022 Durstenfeld MS et al. *JAMA Network Open*.
